# The indispensable N-terminal half of eIF3j/HCR1 co-operates with its structurally conserved binding partner eIF3b/PRT1-RRM and eIF1A in stringent AUG selection

**DOI:** 10.1016/j.jmb.2009.12.047

**Published:** 2010-01-11

**Authors:** Latifa ElAntak, Susan Wagner, Anna Herrmannová, Martina Karásková, Edit Rutkai, Peter J. Lukavsky, Leoš Valášek

**Affiliations:** 1 MRC-Laboratory of Molecular Biology, Structural Studies Division, Hills Road, Cambridge, CB2 2QH, England; 2 Laboratory of Regulation of Gene Expression, Institute of Microbiology AVCR, v.v.i., Videnska 1083, Prague, 142 20, the Czech Republic

**Keywords:** translation initiation, AUG recognition, eIF3, eIF1A, NMR

## Abstract

Despite the recent progress in our understanding of the numerous functions of individual subunits of eukaryotic translation initiation factor 3 (eIF3), there is still only little known on the molecular level. Using NMR spectroscopy, we determined the first solution structure of an interaction between eIF3 subunits. We revealed that a conserved tryptophan residue in the human eIF3j N-terminal acidic domain (NTA) is held in the helix α1 – loop L5 hydrophobic pocket of the human eIF3b-RRM. Mutating the corresponding “pocket” residues in its yeast orthologue reduces cellular growth rate, eliminates eIF3j/HCR1 association with eIF3b/PRT1 *in vitro* and *in vivo*, affects 40S-occupancy of eIF3, and produces a leaky scanning defect indicative of a deregulation of the AUG selection process. Unexpectedly, we found that the N-terminal half (NTD) of eIF3j/HCR1 containing the NTA motif is indispensable and sufficient for wild-type growth of yeast cells. Furthermore, we demonstrate that deletion of either j/HCR1 or its NTD only, or mutating the key tryptophan residues results in the severe leaky scanning phenotype partially suppressible by overexpressed eIF1A, which is thought to stabilize properly formed pre-initiation complexes at the correct start codon. These findings indicate that eIF3j/HCR1 remains associated with the scanning pre-initiation complexes and does not dissociate from the small ribosomal subunit upon mRNA recruitment as previously believed. Finally, we provide further support for earlier mapping of the ribosomal binding site for human eIF3j by identifying specific interactions of eIF3j/HCR1 with small ribosomal proteins RPS2 and RPS23 located in the vicinity of the mRNA entry channel. Taken together we propose that eIF3j/HCR1 closely co-operates with eIF3b/PRT1-RRM and eIF1A on the ribosome to ensure proper formation of the scanning-arrested conformation required for stringent AUG recognition.

## INTRODUCTION

Translation captures the transfer of genetic information stored in DNA into effector molecules, polypeptides. Efficiency and accuracy of the initiation phase of translation is masterminded by numerous proteins called eukaryotic initiation factors (eIFs). Among them, eIF2 associates in its GTP-bound state with methionyl initiator tRNA (Met-tRNA_i_^Met^) to form the ternary complex (TC) that is subsequently recruited to the 40S small ribosomal subunit with help of eIFs 1, 1A, 3, and 5 producing the 43S pre-initiation complex (reviewed in 1 and 2). eIFs 1 and 1A serve to stabilize a conformation that opens the 40S mRNA binding channel 3 required for recruitment of mRNA, bound by the cap-binding complex eIF4F and PABP, in a reaction that is at least in yeast critically stimulated by eIF3 4. In thus formed 48S pre-initiation complex, the 40S subunit is believed to adopt an open/scanning conducive conformation, which enables inspection of successive triplets in the mRNA leader in an ATP-dependent process called scanning that is relatively poorly understood. During this process, eIF5 stimulates partial GTP hydrolysis on eIF2, but the resultant P_i_ is not released until initiation codon – anti-codon base-pairing induces a conformational switch to the closed/scanning arrested form accompanied by displacement of eIF1 (reviewed in 5). This irreversible reaction serves as the decisive rate-limiting step stalling the entire machinery with the AUG start codon placed in the decoding center (P site) of the 40S subunit. eIF1 is responsible for preventing premature engagement with putative start codons whereas eIF1A is believed to stabilize properly formed pre-initiation complexes at the correct start codon. eIF3 also contributes to the latter process via its contacts with eIFs 1, 2 and 5, however, molecular details of its participation are not known 6. After eIF2-GDP release, the 60S large ribosomal subunit can join the 40S-mRNA-Met-tRNA_i_^Met^ pre-initiation complex in a reaction stimulated by a second GTPase, eIF5B. Subunit joining is thought to facilitate ejection of all eIFs but eIF1A 7 and eIF3 8. When eIF5B.GDP dissociates, the 80S initiation complex is ready for elongation.

**Figure 1:**
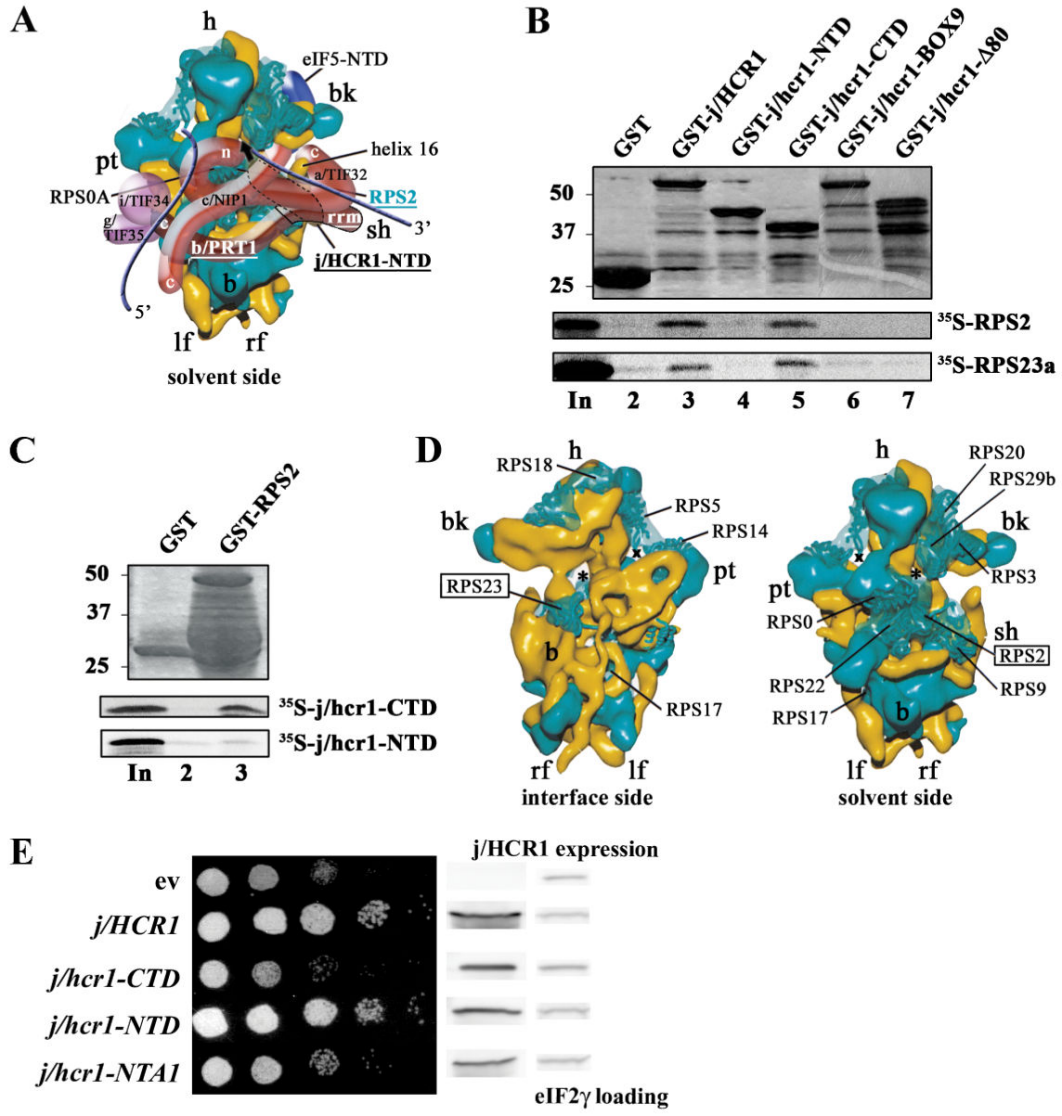
The CTD of j/HCR1 interacts with RPS2 and 23a situated near the 40S mRNA-entry channel but it is dispensable for efficient translation in yeast as opposed to its NTD. (A) Hypothetical location of eIF3 on the solvent side of the *S. cerevisiae* 40S subunit based on Cryo-EM reconstruction (adapted from 14). Protrusion of the CTD of eIF3j into the mRNA entry channel based on 27 is symbolized by a green arrow. The blue lines represent mRNA. (B and C) The j/hcr1-CTD interacts with RPS2 and RPS23a via its KERR motif. (B) Full-length j/HCR1 (lane 3), its N-terminal (lane 4) or C-terminal (lane 5) halves, various mutants (lanes 6 and 7) defined in Fig. 6B fused to GST, and GST alone (lane 2), were tested for binding against ^35^S-labeled wt RPS2 and RPS23; the 10% of input amounts added to each reaction is shown in lane 1 (In). (C) RPS2 fused to GST (lane 3) and GST alone (lane 2) were tested for binding to ^35^S-labeled j/hcr1-CTD and NTD essentially the same as in 1B. (D) Cryo-EM reconstruction of the *S. cerevisiae* 40S subunit (adapted from 28). The 40S subunit is shown from the interface (left) or solvent (right) sides, with RNA segments in yellow and proteins in green. The mRNA entry and exit channels are designated by * and X, respectively. (E) The j/hcr1-NTD is required for wt growth dependent on its intact NTA motif. Transformants of H428 (*j/hcr1Δ*) bearing empty vector; YEp-j/HCR1-DS; YEp-j/hcr1-CTD; YEp-j/hcr1-NTD; and YEp-j/hcr1-NTA1; respectively, were spotted in five serial 10-fold dilutions on SD medium and incubated at 30°C for 1.5 days. The far-right columns contain results of Western analysis of WCEs from the very same strains using anti-j/HCR1 (j/HCR1 expression) and anti-GCD11 (eIF2γ loading) antibodies, respectively.

eIF3 is the most complex initiation factor composed of 6 subunits in yeast *S. cerevisiae* (a/TIF32, b/PRT1, c/NIP1, i/TIF34, g/TIF35, and j/HCR1), all of which have corresponding orthologs in mammalian eIF3 containing additional 7 subunits (d, e, f, h, k, l, m) 9. Given such a complexity, it is not surprising that eIF3 was demonstrated to promote nearly all initiation steps including binding of TC and other eIFs to the 40S subunit, subsequent mRNA recruitment and scanning for AUG recognition (reviewed in 9). These activities are facilitated by other eIFs such as eIF2, eIF1 and eIF5 which make direct contacts with eIF3 and, at least in yeast, occur in the ribosome-free assembly called the Multifactor Complex (MFC) 4; 6; 10; 11; 12; 13. We previously pin-pointed several eIF3 domains that could play a critical role in the MFC-association with the 40S subunit, including the N- and C-terminal domains (NTD and CTD) of c/NIP1 and a/TIF32, and the RNA-recognition motif (RRM) in the NTD of b/PRT1 12; 14. Identification of direct interactions between the NTD of a/TIF32 and the small ribosomal protein RPS0A, and the CTD of a/TIF32 and helices 16–18 of 18S rRNA allowed us to propose that eIF3 associates with the solvent-exposed side of the small subunit 14 (Fig. 1A), as suggested by others for mammalian eIF3 15; 16. In support, we have recently demonstrated that a partial non-lethal deletion of the NTD of a/TIF32 significantly reduced the amounts of 40S-bound MFC components *in vivo* implicating this domain in formation of a critical intermolecular bridge between eIF3 and the 40S subunit 8.

Whereas there is no structural information available on yeast eIF3, whose detailed subunit-interaction map is well defined 10, the recent cryo-EM study of human eIF3 revealed a low resolution particle with a five-lobed architecture 16. The first attempt to unveil details of the spatial arrangement of its subunits and interactions between them suggested that human eIF3 is composed of three relatively stable modules, one of which bears resemblance to the yeast eIF3 core complex 17. Both yeast and mammalian eIF3 were suggested to associate with the 40S subunit via its solvent-exposed side (Fig. 1A) 8; 14; 16. We recently provided the first insight into the molecular nature of eIF3 subdomains by resolving the NMR solution structure of the RRM of human eIF3b (heIF3b) 18. We reported a non-canonical RRM with a negatively charged surface in the β-sheet area contradictory with potential RNA binding activity of typical RRMs. Instead, we found that human eIF3j (heIF3j) interacts with the basic area of heIF3b-RRM, opposite its β-sheet surface, via its N-terminal 69-amino acid peptide and that this interaction promotes heIF3b-RRM recruitment to the 40S subunit.

eIF3b is considered to serve as the major scaffolding eIF3 subunit shown to interact with a, c, g, i, and j in both mammals and yeast 10; 17; 19; 20; 21; 22; 23, clearly illustrating high evolutionary conservation of its structure-organizing role. Indeed, we previously demonstrated that b/PRT1 also interacts with j/HCR1 via its N-terminal RRM domain 23 and this contact was later implicated in the ability of j/HCR1 to stimulate 40S-binding by eIF3. Remarkably, mutating the RNP1 motif of b/PRT1-RRM in *b/prt1-rnp1* was shown to modestly increase leaky scanning suggesting that the RRM of b/PRT1 also contributes to the efficiency of AUG recognition.

j/HCR1 is the only non-essential subunit in yeast 24 believed to stimulate eIF3 binding to the 40S subunit^12^ and to promote 40S ribosome biogenesis 25. Consistently, *in vitro* experiments revealed that heIF3j can bind to the 40S subunit by itself and is required for stable 40S-association of purified eIF3 7; 20; 26. Intriguingly, heIF3j, in the absence of other factors, was also demonstrated to be mutually antagonistic for binding to the 40S subunit with mRNA^7^; ^27^. Furthermore, a mutual exclusivity for heIF3j in 40S subunit binding was also observed with eIF1A^27^. These results together with determination of a position of the heIF3j - CTD in the 40S mRNA entry channel and the ribosomal A site by hydroxyl radical probing 27 suggested that eIF3j may coordinate binding of mRNA and eIFs within the decoding center and thus perhaps influence transitions between scanning conducive and arrested conformations. To gain a full understanding of physiological roles of eIF3j, it is critical to obtain detailed biochemical and structural information of its interactions and to examine their importance in living cells.

Unexpectedly, here we show that the NTD of j/HCR1 is indispensable and sufficient for wild-type (wt) growth. Strikingly, we also found that the deletion of j/HCR1 (or its NTD only) leads to a strong leaky scanning phenotype, indicative of a defect in AUG recognition, partially suppressible by increased gene dosage of eIF1A. These novel results strongly suggest that eIF3j remains bound to scanning ribosomes even after mRNA recruitment. NMR spectroscopic analysis revealed that heIF3j is held via its N-terminal acidic motif (NTA) centered by the conserved tryptophan (Trp52) in a hydrophobic pocket formed by helix α1 (α1) and loop 5 (L5) of the heIF3b-RRM. To our knowledge, this is the first structural insight into molecular interactions within eIF3 from any organism. Mutating these evolutionary conserved determinants in yeast j/HCR1 and b/PRT1 subunits disrupted their direct binding *in vitro* as well as the j/HCR1 association with the MFC but not with 40S subunits *in vivo*. Both j/HCR1 and b/PRT1 mutations resulted in growth phenotypes and imparted severe leaky scanning defects. The b/PRT1-RRM mutation then in addition strongly reduced association of the core eIF3 with 40S subunits suggesting that it forms, either directly or indirectly, an important intermolecular bridge between eIF3 and the small ribosomal subunit. We conclude that the key function of the NTD of j/HCR1 is to co-operate with the RRM of b/PRT1 and eIF1A on the 40S subunit to ensure proper establishment of the scanning-arrested conformation required for stringent AUG recognition.

## RESULTS

### The N-terminal Half of j/HCR1 is Indispensable and Sufficient for Efficient Translation in Yeast

Recent observations made with GST pull-down experiments showed that the last 16 amino acids of heIF3j are required for stable binding of eIF3 to the 40S subunit^20^, and that binding of heIF3j-CTD occurs in the 40S mRNA entry channel 27. Consistent with the latter, using GST pull downs we reproducibly detected weaker but highly specific interactions between the purified j/hcr1-CTD and small ribosomal proteins RPS2 and RPS23 (Fig. 1B, lane 5; and 1C, middle panel) dependent on the last 80 amino acid residues of j/HCR1 and the intact KERR motif (K^205^-x_5_-E^211^R^212^-x_2_-R^215^) (Fig. 1B, lanes 6 and 7), which is conserved between eIF3j and the HCR1-like domain of eIF3a across species (see below)^23^. (None of the remaining 31 small ribosomal proteins interacted with j/HCR1 in this assay.) RPS2 and RPS23 were previously shown to occur on the solvent and interface sides of the mRNA entry channel, respectively 28 (Fig. 1D). Together these findings suggest that the ribosomal binding site of the CTD of eIF3j might have remained evolutionary conserved and that it thus represents an important functional domain of eIF3j.

**Figure 2:**
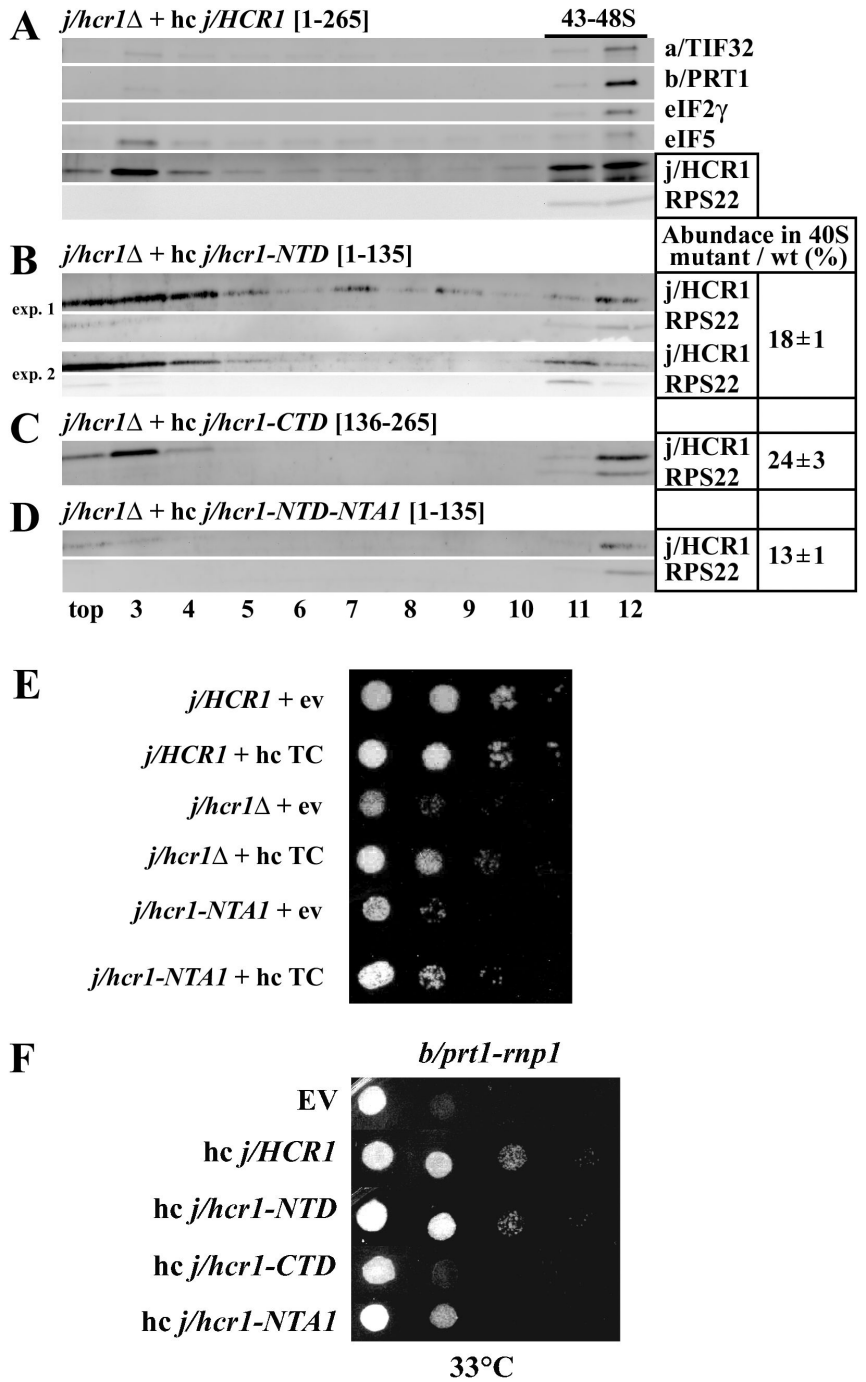
Both the NTD and CTD of j/HCR1 retain intrinsic 40S-binding affinity. (A-D) Transformants of strain H428 (*j/hcr1Δ*) bearing YEp-j/HCR1-DS; YEp-j/hcr1-NTD; YEp-j/hcr1-CTD; and YEp-j/hcr1-NTD-NTA1, respectively, were grown in SD medium at 30°C to an OD_600_ of ~ 1.5 and cross-linked with 2% HCHO prior to harvesting. WCE were prepared and subsequently separated on a 7.5%-30% sucrose gradient by centrifugation at 41,000 rpm for 5 h. The 40S fractions were pooled, resolved on a second gradient, and subjected to Western analysis. First two fractions were combined (top). Proportions of the 40S-bound j/HCR1 proteins relative to the amount of 40S subunits were calculated using NIH ImageJ from two independent experiments. The resulting values obtained with the wt strain were set to 100% and those obtained with mutant strains were expressed as percentages of the wt (SDs are given). (E-F) Genetic evidence that j/HCR1 with intact NTA (or only its NTD) stimulates 40S-binding by eIF3. (E) Overexpression of the TC partially suppresses the Slg^−^ of *j/hcr1Δ* and *j/hcr1-NTA1* mutants. H416 (*j/HCR1*; rows 1 and 2), H428 (*j/hcr1Δ* YEplac181; rows 3 and 4), and SY73 (*j/hcr1Δ* YEp-j/hcr1-NTA1; rows 5 and 6), respectively, were transformed with either empty vector (rows 1, 3, and 5) or the TC overexpressing vector (rows 2, 4, and 6) and the resulting transformants were spotted in four serial 10-fold dilutions on SD medium and incubated at 30°C for 2 days. (F) j/HCR1 with intact NTA (or only its NTD) partially suppresses the Ts^−^ phenotype of *b/prt1-rnp1*. Transformants of the strain H3674 (*b/prt1-rnp1*) 12 bearing empty vector; YEp-j/HCR1-DS; YEp-j/hcr1-NTD; YEp-j/hcr1-CTD; and YEp-j/hcr1-NTA1, respectively, were spotted in four serial 10-fold dilutions on SD medium and incubated at 33°C for 3 days.

To examine this possibility, we first expressed the N- and C-terminal domains of j/HCR1 (defined in Fig. 6B) in the *j/hcr1Δ* strain and tested the resulting transformants for suppression of its slow growth phenotype (Slg^−^). Surprisingly, we found that the CTD of j/HCR1 is dispensable for the wt growth of yeast cells in contrast to its NTD, the deletion of which phenocopied the Slg^−^ phenotype of *j/HCR1* deletion (Fig. 1E, 4^th^ vs. 3^rd^ rows). (Both truncated proteins, as well as other j/HCR1 mutants mentioned below, had to be tested from high copy vectors due to their decreased stability. In this arrangement, their expression levels were about 3-fold higher than the physiological level and similar to the level of overexpressed wt j/HCR1 that does not produce any phenotypes (Fig. 1E; and data not shown)). This finding implies that the NTD of j/HCR1 should be able to associate with the 40S subunit independently of its CTD. To test this we employed formaldehyde cross-linking method followed by resedimentation of the 40S fractions on a second gradient to minimize trailing of non-cross-linked factors into 40S fractions. It is worth mentioning that this method provides the best available approximation of the native 43S/48S pre-initiation complexes composition *in vivo* 29. As shown in Fig. 2A – C, both the j/hcr1-NTD and j/hcr1-CTD retained similar ~20 % of wt affinity towards the 40S subunit. (Bands in the upper fractions after resedimentation most likely represent j/HCR1 proteins not properly crosslinked to pre-initiation complexes *in vivo* that dropped off during two consecutive high velocity centrifugations.) Taken into account the non-equilibrium character of this assay, the given percentages are only relative numbers and in principle suggest that both j/HCR1 halves show less stable binding to 40S subunits under these conditions than the full-length protein. In fact, since the j/HCR1-NTD fully supports growth of *j/hcr1Δ* cells, it seems likely that in the living cells it associates with 40S subunits more efficiently. To learn whether the j/HCR1-NTD–40S interaction is bridged by eIF3, we examined 40S-binding of the j/hcr1-NTD bearing a specific *NTA1* mutation, which, as described in detail below, destroys a direct j/HCR1 – b/PRT1 interaction and completely diminishes j/HCR1 association with the MFC *in vivo* (Figs. 6C and 7B). As shown in Fig. 2D, the j/hcr1-NTD-NTA1 mutant still associated with 40S subunits, albeit with a reduced affinity by ~ 30% compared to the j/hcr1-NTD. Together, these experiments indicate that both halves of j/HCR1 possess intrinsic 40S-binding affinity that is additive and further strengthened by j/HCR1 contacts with 40S-bound eIF3.

Deletion of *j/HCR1* was previously shown to reduce amounts of 40S-bound eIF3 12. We next wished to show that the wt-like behaving j/hcr1-NTD is also fully capable to support eIF3 loading onto the 40S subunit. However, the differences in the amounts of eIFs associated with 40S subunits between wt and *j/hcr1Δ* cells were somewhat smaller when compared to the previous study. Because this discrepancy is still under examination, we could not conclusively address this question here. Nevertheless, we made two genetic observations supporting the idea that at least part of the *j/hcr1Δ* growth defect could be associated with the reduced eIF3-binding to the 40S subunit and that the j/hcr1-NTD can fully substitute full length j/HCR1 in this respect: i) overexpression of all three eIF2 subunits and tRNA_i_^Met^ (hc TC), previously shown to stimulate j/HCR1-independent 40S-binding of eIF3 4; 12, partially suppressed the Slg^−^ of *j/hcr1Δ* cells (Fig. 2E, 4^th^ vs. 3^rd^ rows); and ii) overexpression of the j/hcr1-NTD but not the j/hcr1-CTD suppressed the Slg^−^ of the *b/prt1-rnp1* mutant to the same degree as full length wt j/HCR1 (Fig. 2F, rows 3 and 4). The *b/prt1-rnp1* mutation was previously shown to affect eIF3-binding to the 40S subunit in a manner partially suppressible by high copy j/HCR1 (see also below) 12. Interestingly, J. Lorsch and colleagues also did not observe any effect on increased binding of eIF3 (containing only trace amounts of endogenous j/HCR1) to 43S complexes by addition of saturating amounts of separately purified j/HCR1 *in vitro* (J. Lorsch, personal communication, 2009). Taken together, this suggests that in yeast the effect of j/HCR1 on binding of the rest of eIF3 to 40S subunits may be more subtle than it was believed.

### Genetic Evidence that the NTD of j/HCR1 Promotes Proper Selection of the AUG Start Codon in Co-operation with eIF1A

The fact that heIF3j was suggested to govern access to the mRNA entry channel and influence mRNA-40S subunit association during scanning and AUG recognition 27 prompted us to examine the stringency of AUG selection in the *j/hcr1Δ* cells. Mainly we were interested in assaying a leaky scanning defect that might suggest that the scanning pre-initiation complexes have a reduced ability to switch from the scanning-conducive conformation to scanning-arrested conformation when the start codon enters the P site 30.

To investigate this, we took advantage of a reinitiation mechanism of *GCN4* translational control that can be used as an experimental tool to monitor various translational steps. Translation of *GCN4* mRNA is repressed in nutrient replete cells by the last three of total four short upstream ORFs in its leader. Under starvation conditions, the concentration of TC is reduced and as a result, a fraction of 40S subunits scanning downstream after terminating at first reinitiation-permissive uORF1 rebind TC only after bypassing inhibitory uORFs 2–4 and then reinitiate at *GCN4* 31. Leaky scanning leads to skipping over AUG of uORF1 by scanning ribosomes, which subsequently initiate at downstream inhibitory uORFs preventing the cells to derepress *GCN4* translation under starvation conditions. This phenotype is called Gcn^−^ (general control nonderepressible) and is characterized by the sensitivity of mutant cells to 3-aminotriazole (3-AT), an inhibitor of the *HIS3* product.

**Figure. 3:**
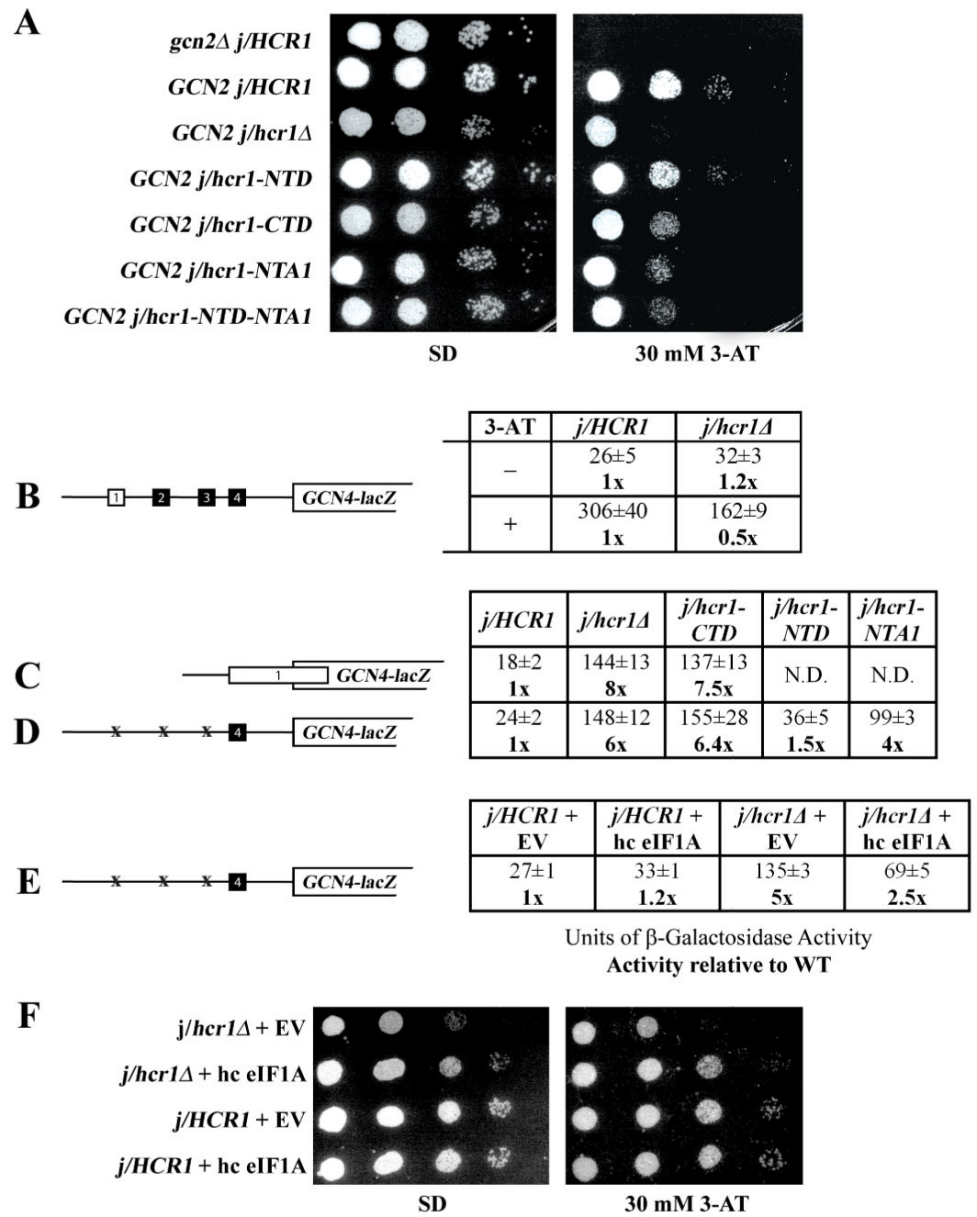
Genetic evidence that the deletion of the j/hcr1-NTD (or the NTA1 mutation) prevents derepression of GCN4 translation during starvation as a result of leaky scanning partially suppressible by high copy eIF1A. (A) *j/hcr1Δ* imparts a Gcn^−^ phenotype implicating j/HCR1 in *GCN4* translational control. H418 (*gcn2Δ j/HCR1;* row 1) and the H428 (*GCN2 j/hcr1Δ*) transformants bearing YEp-j/HCR1 (row 2); YEplac181 (row 3); YEp-j/hcr1-NTD (row 4); YEp-j/hcr1-CTD (row 5); YEp-j/hcr1-NTA1 (row 6); and YEp-j/hcr1-NTD-NTA1 (row 7), respectively, were spotted in four serial 10-fold dilutions on SD (left panel) or SD containing 30 mM 3-AT (right panel) and then incubated at 30°C for 2 and 3 days, respectively. (B) *j/hcr1Δ* prevents full derepression of *GCN4-lacZ* expression upon starvation. Isogenic H416 (*GCN2 j/HCR1*) and H428 were transformed with p180, grown in minimal media for 6 hours and the β-galactosidase activities were measured in the WCEs and expressed in units of nmol of o-nitrophenyl-b-D-galactopyranoside hydrolyzed per min per mg of protein. To induce *GCN4-lacZ* expression, transformants grown at the minimal media for 2 hrs were treated with 10 mM 3-AT for 6 hrs. The table gives mean values and standard deviations obtained from at least 6 independent measurements with three independent transformants, and activity in *j/hcr1Δ* relative to wt, respectively. (C - D) Deletion of *j/hcr1Δ* or its NTD only dramatically increases leaky scanning. H428 transformants bearing YEp-j/HCR1; YEplac181; YEp-j/hcr1-CTD; YEp-j/hcr1-NTD; and YEp-j/hcr1-NTA1, respectively, were transformed with pM226 (C) and plig102-3 (D), respectively, and analyzed as in (B), except that they were not treated with 3-AT. (E) Overexpression of eIF1A partially suppresses the leaky scanning defect of *j/hcr1Δ*. Strains H416 and H428 transformed with empty vector and YEpTIF11 (eIF1A), respectively, were transformed with plig102-3 and analyzed as in (D). (F) Overexpression of eIF1A partially suppresses the Slg^−^ and Gcn^−^ phenotypes of *j/hcr1Δ*. Strains H428 and H416 transformed with empty vector and YEpTIF11, respectively, were spotted in four serial 10-fold dilutions on SD or SD + 30 mM 3-AT and incubated at 30°C for 2 or 4 days.

We found that *j/hcr1Δ GCN2*^+^ cells exhibit significant sensitivity to 3-AT (Fig. 3A, row 3) that was further illustrated by ~ 50% reduction in derepression of the wt *GCN4-lacZ* reporter in response to 3-AT compared to wt *j/HCR1*^+^ (Fig. 3B, “+”). Strikingly, examination of a *GCN4–lacZ* construct in which uORF1 is elongated and overlaps the beginning of *GCN4* revealed ~8-fold increase in *GCN4–lacZ* expression in *j/hcr1Δ* cells (Fig. 3C, column 2). Similarly, ~6-fold increase in *GCN4–lacZ* expression was also detected from a construct containing solitary uORF4 (Fig. 3D, column 2) that allows only a negligible level of reinitiation 8; 32. These results thus strongly suggest that deletion of *j/HCR1* impairs *GCN4* translational control by allowing a large fraction of pre-initiation complexes scanning from the cap to leaky scan at AUG of uORF1. Furthermore, the cells expressing the NTD-less j/hcr1-CTD also displayed 3-AT sensitivity (Fig. 3A, row 5) and increased the *GCN4–lacZ* expression with constructs monitoring leaky scanning (Fig. 3C and D, column 3) by ~ 7-fold, as opposed to those expressing the CTD-less j/hcr1-NTD that increased leaky scanning only by a small margin (Fig. 3D, column 4). Hence these results clearly suggest that the NTD is for the most part responsible for the j/HCR1 contribution to the stringent AUG selection.

eIF1A was shown to functionally interact with heIF3j 27 and is thought to facilitate pausing of the scanning pre-initiation complexes at the correct start codon long enough to proceed with downstream initiation events, in other words to prevent leaky scanning 5; 30. Accordingly, we observed that overexpression of eIF1A partially suppressed both Slg- and Gcn- phenotypes of *j/hcr1Δ* (Fig. 3F, row 2) and, most importantly, reduced leaky scanning over uORF4 by ~ 50% (Fig. 3E, last column). Taken together, we propose that the NTD of j/HCR1 communicates with eIF1A during scanning and promotes the eIF1A role in inducing the smooth transition to the closed/scanning-arrested conformation upon AUG recognition.

### The overall structure of the heIF3b-RRM_170-274_ - heIF3j_35-69_ complex

**Figure. 4:**
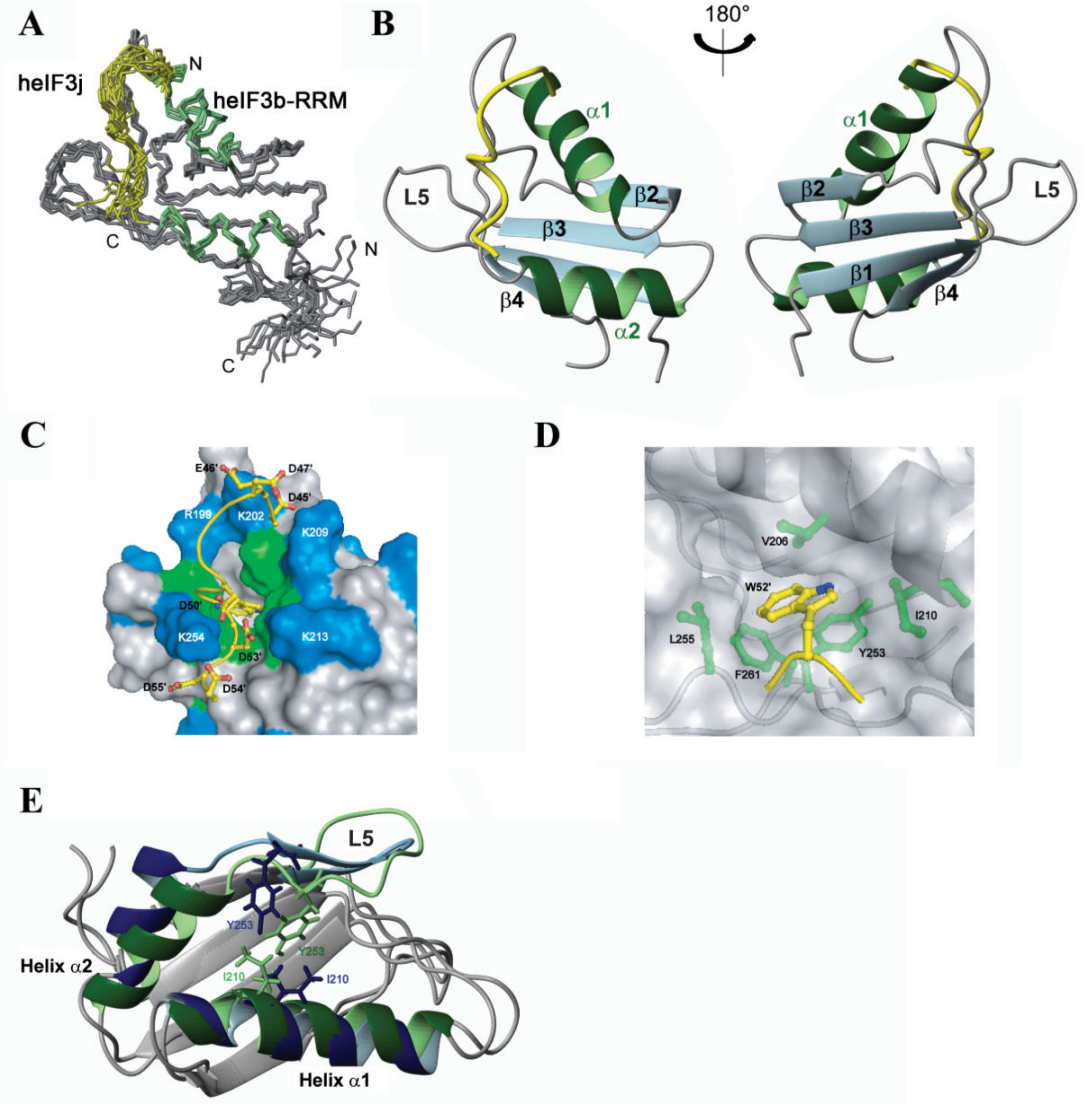
Structure of the heIF3b-RRM - heIF3j complex. (A) NMR ensemble of heIF3b-RRM - heIF3j peptide complex. The 10 lowest-energy structures between heIF3b-RRM_179-274_ (in grey and green) and heIF3j_45-55_ (in yellow) are shown. The structures were fit using the backbone atoms C’, C^α^, and N of residues 184-264 of heIF3b-RRM and residues 45-55 of heIF3j. (B) Ribbon model for the lowest-energy conformer of the heIF3b-RRM (in grey and green)/heIF3j (in yellow) complex. Secondary structure elements of heIF3b-RRM are labeled. (C) Surface representation of the contacts between heIF3j peptide and heIF3b-RRM. Green and blue surfaces indicate hydrophobic and basic (labeled) heIF3b-RRM residues, respectively. heIF3j peptide is shown as a ribbon ball-and-stick representation, and most of its residues are numbered with primed numbers. The lowest-energy structure of heIF3b-RRM bound to heIF3j peptide is shown. (D) Close-up view of the hydrophobic pocket binding the heIF3j peptide. The heIF3b-RRM is displayed as grayish semitransparent solvent-accessible surface with labeled hydrophobic side chains in green shown below the surface. These residues form the walls of the hydrophobic pocket in which the aromatic ring of W52′ of the heIF3j peptide in yellow is inserted (residues 51-53 only). (E) Comparison of NMR structures of free and heIF3j-bound heIF3b-RRM. The two structures are represented as ribbon models with helices α1 and α2 and loop L5 colored in green for heIF3j-bound and in blue for free heIF3b-RRM. The side chains of Y253 and I210 are shown in stick representation using the same coloring scheme to highlight closer contacts participating to a more compact conformation of heIF3b-RRM when bound to heIF3j.

To gain deeper insight into the collaboration between j and b subunits of eIF3, we determined the solution structure of heIF3b-RRM_170-274_ in complex with heIF3j_35-69_ by high-resolution NMR spectroscopy. Stereo views of the 10 lowest-energy structures (Fig. S1) and the structural statistics (Table 1) demonstrate a well-defined complex structure with low pairwise rmsd values of 1.19 ± 0.4 Å for heavy atoms corresponding to residues 180-266 and 45-55 of heIF3b-RRM and heIF3j, respectively. The structure of heIF3b-RRM in the complex presents a typical RRM fold consisting of two perpendicular α helices packed against a four-stranded antiparallel β sheet (Fig. 4A and B) 33; 34. The heIF3j N-terminal peptide is unstructured in the free form (data not shown) and binds heIF3b-RRM in an extended conformation on the surface opposite to the β sheet area of heIF3b-RRM (Fig. 4B). The heIF3j binding surface on heIF3b-RRM comprises helix α1 and the loop L5. Eleven of thirty-five residues (Asp45-Asp55) of the negatively charged heIF3j_35-69_ peptide, which are part of its NTA, directly contact heIF3b-RRM (Fig. 4C). The total buried surface area of the protein-protein interface is 1128.4 Å^2^ (501.6 Å^2^ on the heIF3b-RRM and 626.8 Å^2^ on the heIF3j peptide). The heIF3b-RRM interaction surface is characterized by positively charged residues from helix α1 (Arg199, Lys202, Lys209, Lys213) and loop L5 (Lys254) that complement and position the negatively charged heIF3j_35-69_ peptide (Fig. 4C). These interactions are illustrated by intermolecular NOEs which bring Lys254-Hε into close contact with Asp54-Hα, Lys254-Hγ with Asp54-Hβ, Lys202-Hε with Val48-Hγ, and Lys209-Hβ with Asp45-Hα, respectively (Fig. S2B). At the center of the NTA resides the highly conserved Trp52 which establishes a series of close contacts with heIF3b-RRM (Fig. 4C). The indole ring of Trp52 fills a hydrophobic pocket formed by residues from helix α1 (Leu203, Val206, Ile210), and L5 (Tyr253, Leu255, Phe261) (Fig. 4D). Intermolecular NOEs involving Trp52 ring atoms, such as Hδ1 and Hζ2, with Ile210 and Ile207 as well as with Tyr253 and Leu255 represent key contacts for defining the hydrophobic pocket around Trp52 (Fig. S2B). Binding of heIF3j unfolds the β–hairpin in loop L5 and induces a rearrangement of helix α1 and loop L5 as compared to the unbound heIF3b-RRM (Fig. 4E). This creates a more compact heIF3b-RRM conformation illustrated by a closer contact between Ile210 and Tyr253 which deepens the binding pocket filled by Trp52 of heIF3j.

### Mutational Analysis of the heIF3b-RRM–heIF3j-NTA Interaction

**Figure. 5:**
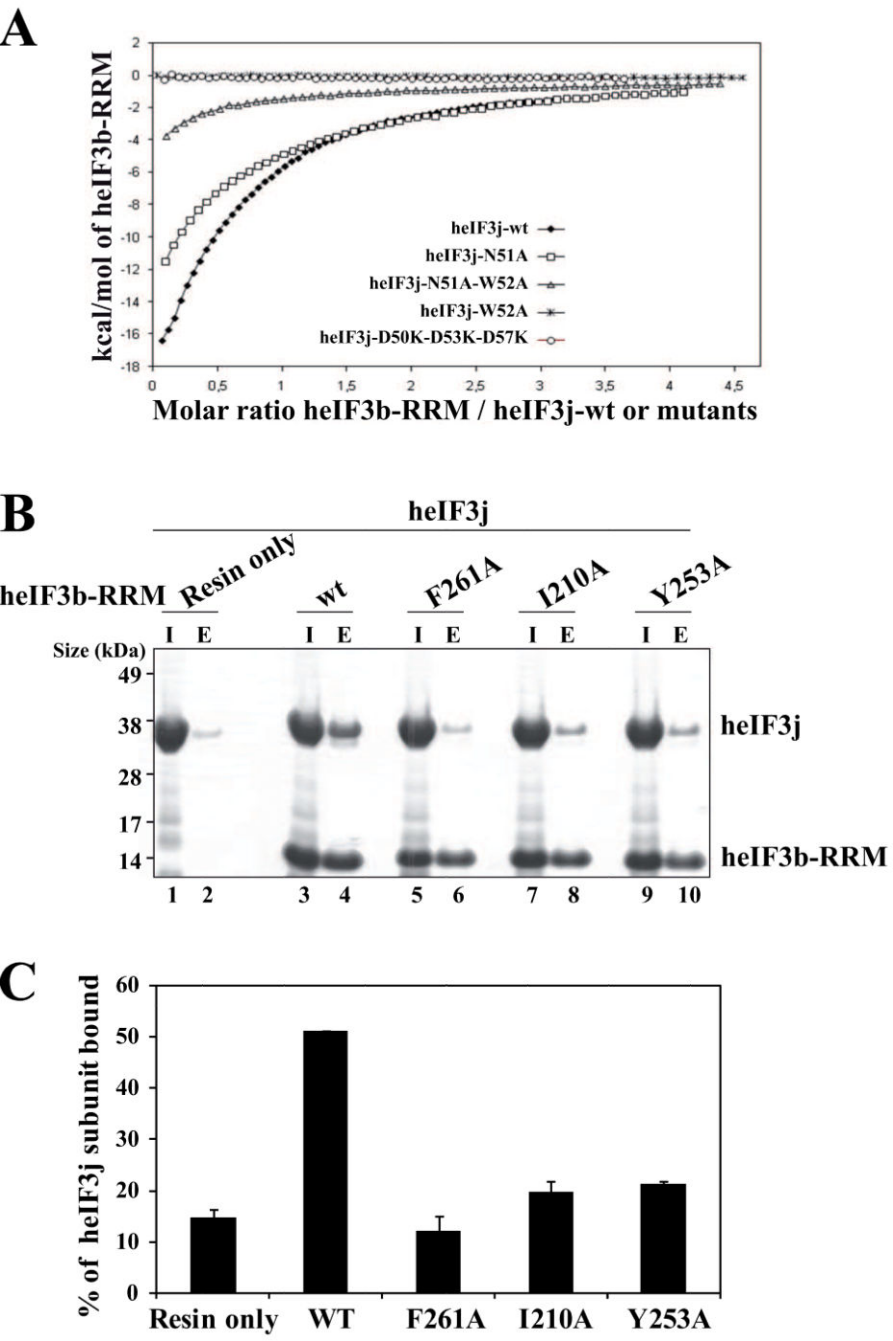
Mutational analysis of the heIF3b-RRM - heIF3j-NTA interaction. (A) Isothermal calorimetric titration of wt and mutant heIF3j with heIF3b-RRM. The panel shows the fitted binding isotherms. The data points were obtained by integration of heat signals plotted against the molar ratio of heIF3b-RRM to wt or mutant heIF3j in the reaction cell. The solid line represents a calculated curve using the best fit parameters obtained by a nonlinear least squares fit. The heIF3j construct used for each experiment is indicated in the panel. (B) Histidine pull-down assays using His_6_-tagged wt or mutant heIF3b-RRM and untagged heIF3j. SDS-PAGE analysis of the input and eluted fractions (I and E) from the pull-down experiments where the untagged heIF3j subunit was used with wt or mutant (F261A, I210A, Y253A) heIF3b-RRM as well as alone (Resin only) as a control. (C) Quantification of heIF3j fraction bound to heIF3b-RRM by analyzing the band intensity of the eluted fraction compared to the same band in the input fraction. Error bars represent the standard deviation between two individual experiments.

To assess the relative contribution of key residues for heIF3b-RRM - heIF3j complex formation, we mutated several important interface residues. Binding of four heIF3j mutants (heIF3j-N51A, heIF3j-N51A-W52A, heIF3j-W52A, heIF3j-D50K-D53K-D57K) to heIF3b-RRM was examined using Isothermal Titration Calorimetry (ITC). The heIF3j mutants displayed significantly lower affinities than wt heIF3j (Kd = 20.3+/−0.4 μM). In this assay, we were unable to detect any heIF3b-RRM binding to heIF3j-W52A, heIF3j-N51A-W52A and heIF3j-D50K-D53K-D57K indicating Kd values larger than 10 mM, whereas heIF3j-N51A bound with a lower Kd of 55±0.3 μM (Fig. 5A). These results agree with our complex structure showing that heIF3j-Trp52 makes crucial hydrophobic contributions to the heIF3b-RRM binding and that surrounding negatively charged heIF3j-NTA residues further stabilize complex formation (Fig. 4C).

We also performed histidine pull-down assays using three heIF3b-RRM mutants (heIF3b-RRM-F261A, heIF3b-RRM-I210A and heIF3b-RRM-Y253A) to assess contributions of hydrophobic heIF3b-RRM residues to heIF3j binding. All three heIF3b-RRM mutants displayed significantly reduced binding compared to the wt heIF3b-RRM validating the role of the heIF3b-RRM hydrophobic pocket in heIF3j recognition (Fig. 5B and C). Interestingly, hydrophobic amino acid residues in positions Leu203, Val206, Ile210, Tyr253, and Phe261 are highly conserved among eIF3b-RRMs from other species indicating that the heIF3b-RRM - heIF3j recognition mode is preserved in other organisms (Fig. S3).

### Molecular Determinants of the eIF3j – eIF3b-RRM Interaction are Conserved Throughout Evolution

**Figure. 6:**
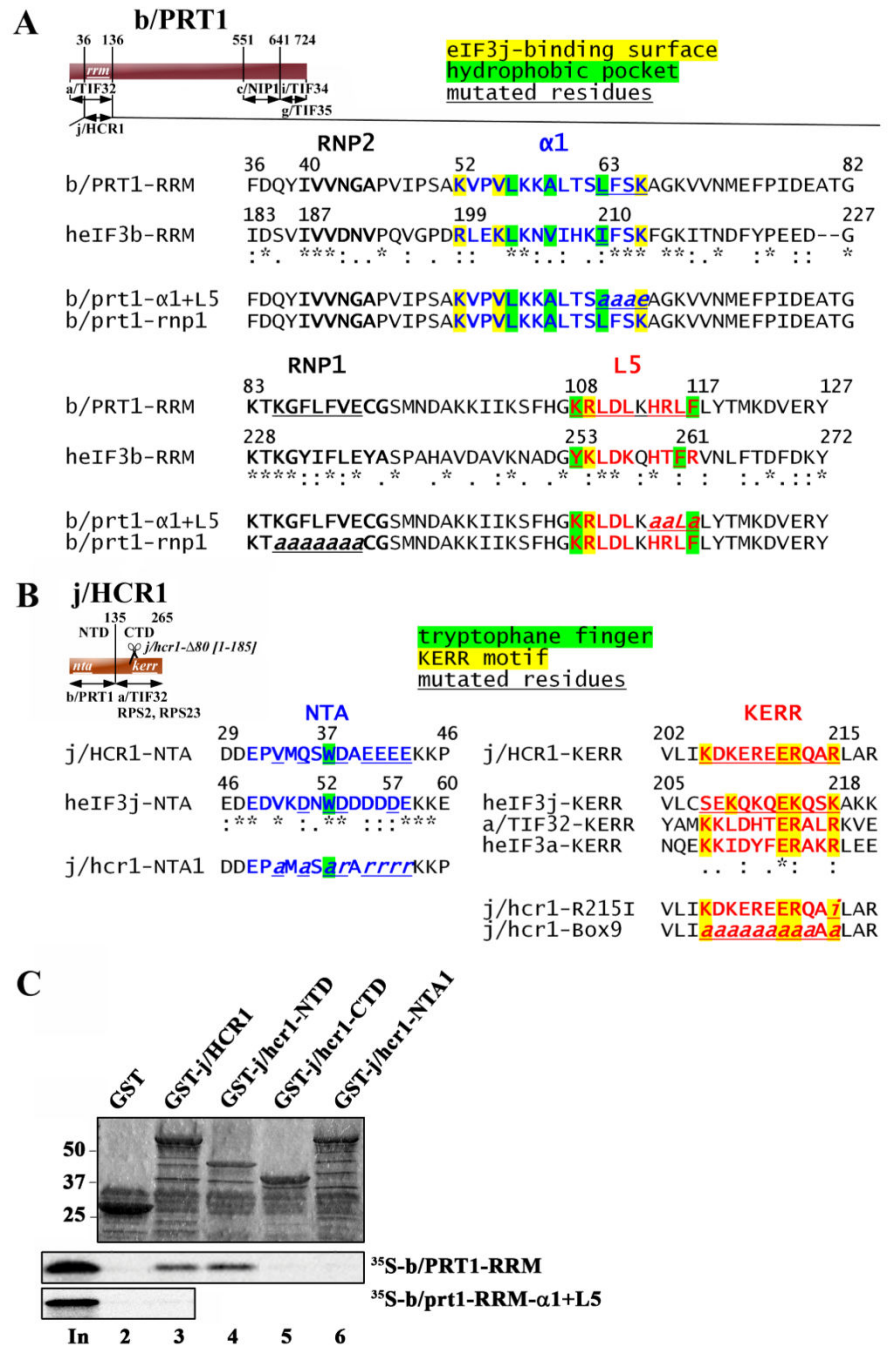
Molecular determinants of the eIF3j–eIF3b-RRM interaction are evolutionary conserved. (A) Schematic of b/PRT1 showing the position of the RNA recognition motif (*rrm*). Arrows delimit minimal binding domains for the indicated proteins. Positions of both RNPs (black), helix α1 (blue) and loop L5 (red) are indicated above the sequences aligned using the GCG Analysis Program. Residues corresponding to the heIF3j binding surface are highlighted in yellow, residues forming the hydrophobic pocket in green. Underlined are b/PRT1 residues that were subjected to site-directed mutagenesis in this or previous studies 12. (B) Same as in (A) except that the schematic of j/HCR1 is shown with locations of the N-terminal acid motif (*nta*), the C-terminal KERR motif (*kerr*), and the C-terminal truncation (*Δ80*). Sequences surrounding the NTA and KERR motifs of yeast and human eIF3j or eIF3a, respectively, are indicated. Underlined are j/HCR1 resides that were subjected to site-directed mutagenesis in this or previous studies 23. The human Trp52 and the corresponding yeast Trp37 are highlighted in green; the key residues of the KERR motif in yellow. (C) Full-length j/HCR1 (lane 3), its N-terminal (lane 4) or C-terminal (lane 5) halves, the *NTA1* mutant (lane 6) fused to GST, and GST alone (lane 2), were tested for binding to ^35^S-labeled wt b/prt1-RRM [1-136] and b/prt1-rrm-α1+L5; the 10% of input amounts added to each reaction is shown in lane 1 (In).

To investigate whether the critical determinants of the eIF3b – eIF3j interaction in yeast are similar in nature to those in humans, we first fused both halves of j/HCR1 in j/hcr1-NTD (1-135) and j/hcr1-CTD (136-265) (Fig. 6B) with the GST moiety and showed that the NTD but not the CTD of j/HCR1 specifically interacts with the [^35^S]-labeled fragment comprising the b/prt1-RRM (Fig. 6C, lanes 4 versus 5). We then substituted the Trp37 residue corresponding to the key Trp52 of heIF3j and several surrounding acidic residues from its NTA with alanines or amino acids with the opposite charge (Fig. 6B). The resulting *j/hcr1-NTA1* mutation completely abolished binding to radiolabeled b/prt1-RRM (Fig. 6C, lane 6). Similarly, alanine and opposite charge substitutions of the b/PRT1-RRM residues corresponding to critical residues in helix α1 and loop L5 of heIF3b in *b/prt1-α1*+*L5* (Fig. 6A) eliminated the interaction with GST-j/HCR1 (Fig. 6C, row 3).

**Figure. 7:**
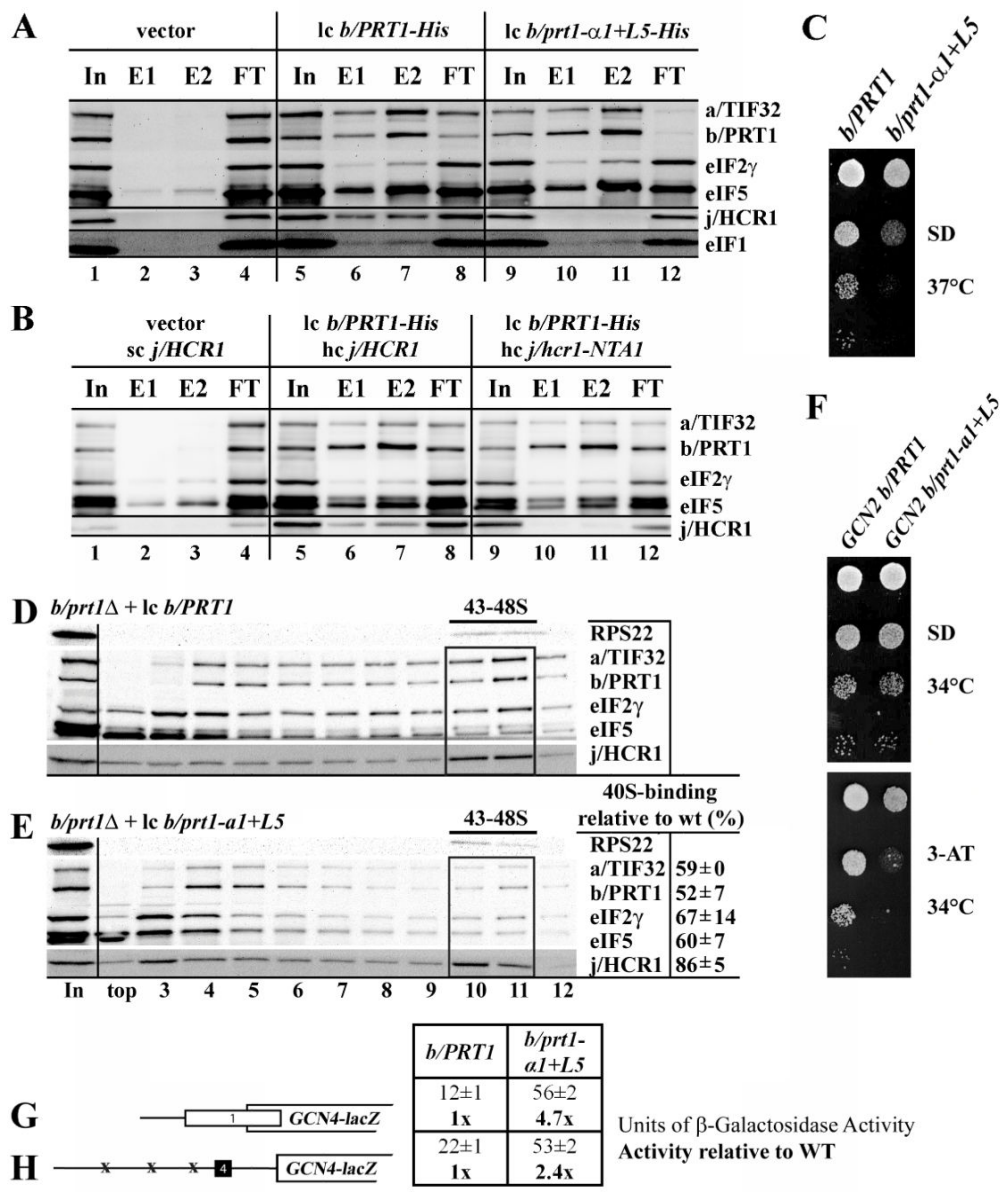
Destroying the hydrophobic pocket of the RRM of b/PRT1 prevents j/HCR1-association with eIF3 *in vivo*, reduces eIF3-binding to 40S subunits, and increases leaky scanning. (A – B) The NTA of j/HCR1 and α1 and L5 of b/PRT1-RRM are critically required for j/HCR1 association with the MFC *in vivo*. (A) WCEs were prepared from H425 (*b/prt1Δ*) bearing untagged b/PRT1/PRT1 (lanes 1 to 4), and H425 transformants with pRS-b/PRT1-His (lanes 5 to 8) and pRS-b/prt1-L5+α1-His (lanes 9 to 12) from which the untagged b/PRT1 was evicted on 5-FOA, respectively, were incubated with Ni^2+^ silica resin, and the bound proteins were eluted and subjected to Western blot analysis with antibodies indicated on the right-handed side of individual strips. Lanes 1, 5 and 9 contained 5% of the input WCEs (In); lanes 2, 6, and 10 contained 30% of fractions eluted from the resin (E1); lanes 3, 7, and 11 contained 60% of the same fractions (E2); and lanes 4, 8, and 12 contained 5% of the flow through (FT). (B) WCEs prepared from double transformants of H428 (*j/hcr1Δ*) bearing pRS315 and YCp-j/HCR1-DS-U (lanes 1 to 4); pRS-b/PRT1-His and YEp-j/HCR1-DS-U (lanes 5 to 8), or pRS-b/PRT1-His and YEp-j/hcr1-NTA1-U (lanes 9 to 12), respectively, were analyzed as in (A). (C – E) Mutating the hydrophobic pocket of the RRM of b/PRT1 results in the Slg- phenotype and strongly affects 40S-association of eIF3. (C) H425 transformants as in (A) were spotted in four serial 10-fold dilutions on SD medium and incubated at 37°C for 2 days. (D - E) H425 transformants as in (A) were grown in SD medium at 37°C to an OD_600_ of ~ 1.5 and analyzed as in Figure 2A-D except that the resedimentation protocol was not applied. Mean proportions of the total proteins found in fractions 10-11 were calculated using NIH ImageJ from two independent experiments. The resulting values obtained with the indicated eIFs with the wt strain were set to 100% and those obtained with the mutant strain were expressed as percentages of the wt (SDs are given). (F - H) The *b/prt1-α1*+*L5* mutation impairs derepression of GCN4 translation during starvation as a result of leaky scanning. (F) *b/prt1-α1*+*L5* imparts the Gcn^−^ phenotype. YAH06 (*GCN2 b/prt1*Δ) transformants carrying pRS-b/PRT1-His and pRS-b/prt1-L5+α1-His, respectively, were spotted in four serial 10-fold dilutions on SD (upper panel) or SD containing 30 mM 3-AT (bottom) and then incubated at 34°C for 2 and 3 days, respectively. (G and H) *b/prt1-α1*+*L5* strongly increases leaky scanning. YAH06 transformants as in (F) were transformed with pM226 (G) and plig102-3 (H), respectively, and analyzed as in Fig. 3C and D.

To further determine whether disrupting this contact will prevent j/HCR1 association with eIF3 *in vivo*, we analyzed formation of the entire eIF3-containing MFC in yeast cells by Ni^2+^-chelation chromatography using His_8_-tagged *b/PRT1* as bait. As reported previously 25, a fraction of a/TIF32, j/HCR1, eIF2, eIF5, and eIF1 copurified specifically with wt b/PRT1-His but not with its untagged version (Fig. 7A, lanes 5 - 8 vs. 1 - 4). In sharp contrast, the *b/prt1-α1*+*L5* mutation (LFSK63-66AAAE_HRLF114-117AALA; Fig. 6A) specifically eliminated association of only j/HCR1 (Fig. 7A, lanes 9 - 12). Similarly, the *j/hcr1-NTA1* mutation (V33A_Q35A_W37A_D38R_EEEE40-43RRRR; Fig. 6B) diminished binding of j/HCR1 to the purified b/PRT1-His complex (Fig. 7B, lanes 9 – 12 vs. 5 – 8).

Finally, disrupting the j/HCR1-NTA–b/PRT1-RRM interaction by the *j/hcr1-NTA1* and *b/prt1-α1*+*L5* mutations, respectively, in living cells resulted in the Slg^−^ phenotype (Fig. 1E, row 5; and 7C, row 2). No growth phenotypes were observed with less extensive mutations in the *HCR1-NTA1* motif or when α1 and L5 of *PRT1* were mutated separately arguing against general refolding problems of these two motifs (data not shown).

### The b/prt1-α1+L5 Mutation Strongly Affects 40S-association of eIF3

As mentioned above, the *b/prt1-rnp1* mutation substituting the conserved residues of the RNP1 motif forming the β3 strand of the four-stranded antiparallel β-sheet with a stretch of alanines (Fig. 6A) eliminated j/HCR1 from the MFC 12 and severely affected binding of the mutant form of eIF3 with the 40S subunit^12^. While this mutation occurs on the opposite side to that directly engaged in interacting with j/HCR1, based on our NMR structure 18 it changes two amino acids in heIF3b-RRM (I233 and L235) and presumably also in b/PRT1-RRM at equivalent positions (L88 and V90), which contribute to the hydrophobic core of the RRM fold. It is therefore conceivable that these substitutions interfere with the proper folding and thus the *b/prt1-rnp1* effects cannot be directly related to the specific loss of contacts that the RRM of b/PRT1 makes. This assumption gains support from our observation that the Slg^−^ of *b/prt1-rnp1* but not of *b/prt1-α1*+*L5* can be partially suppressed by high copy j/HCR1 by mass action (Fig. 2F and data not shown). It is understandable that the elevated protein mass can drive establishment of only that interaction, whose key determinants remain preserved in spite of potential destabilization of the protein fold.

To examine if the *b/prt1-α1*+*L5* mutation specifically disrupting the direct b/PRT1-RRM–j/HCR1 contact also affects 40S-association of mutant eIF3, we measured binding of selected eIF3 subunits and other MFC components to 40S subunits by formaldehyde cross-linking. We observed a relative ~45% decrease in the amounts of selected eIF3 subunits associated with 40S subunits in whole-cell extracts (WCEs) obtained from the *b/prt1-α1*+*L5* cells compared to the wt control (Fig. 7D and E, fractions 10 and 11). Similar reductions were also observed for eIF5 (~40%) and eIF2 (~35%). In keeping with our previous finding with *b/prt1-rnp1* 12, amounts of the 40S-associated j/HCR1 were reduced only marginally (~15%). Since, under the conditions of our experiments, the data suggest that j/HCR1 does not play a key role in eIF3 association with the 40S subunit, this dramatic defect cannot be fully attributable to the loss of the b/PRT1-RRM-j/HCR1 interaction implying that α1 and L5 residues are most probably directly involved in bridging the 40S-eIF3 contact in yeast. Nevertheless, our observations that the *NTA1* mutation, which did not affect the 40S-eIF3 interaction (data not shown), failed to suppress the Slg^−^ of *b/prt1-rnp1* (Fig. 2F, last row) and its own Slg^−^ was found partial suppressible by a plasmid overexpressing all three eIF2 subunits and tRNA_i_^Met^ (hc TC) (Fig. 2E, last two rows) seem to indicate that it does compromise the mild stimulatory effect of j/HCR1 on 40S-binding by eIF3.

### The j/HCR1 – b/PRT1-RRM Interaction Prevents Leaky Scanning over the AUG Start Codon

Our finding that the deletion of the NTD of j/HCR1 produced severe leaky scanning (Fig. 3C and D) and the fact that a modest leaky scanning defect was also observed with *b/prt1-rnp1*^12^ provoked us to test if disrupting the specific contact between j/HCR1 and b/PRT1-RRM will affect the level of leaky scanning in the mutant cells. Indeed, *j/hcr1-NTA1* and *j/hcr1-NTD-NTA1* mutants displayed 3-AT sensitivity (Fig. 3A, last two rows) and greatly increased leaky scanning over uORF4 by ~4-fold (Fig. 3D, column 5). Similarly, the *b/prt1-α1*+*L5* mutant showed reduced growth rate in the presence of 3-AT even at 34°C (Fig. 7F) and also significantly increased leaky scanning over elongated uORF1 by ~4.7-fold (Fig. 7G) and over uORF4 by ~2.4-fold (Fig. 7H). Hence, these data strongly suggest that the evolutionary conserved b/PRT1-RRM – j/HCR1-NTA interaction ensures tight control over the stringent selection of the proper AUG start codon.

## DISCUSSION

### The NMR solution structure of the heIF3b-RRM–heIF3j-NTA interaction

eIF3 plays critical roles in virtually all stages of translation initiation, during reinitiation, post-termination ribosomal recycling, and nonsense-mediated decay pathway 8; 9; 35; 36. In order to understand how the numerous functions of eIF3 are encoded in its conserved subunits and their interactions, high-resolution structural studies of protein-protein interactions of eIF3 subunits are imminent. Using NMR spectroscopy, we revealed a first structure of an interaction among eIF3 subunits, between heIF3b-RRM and heIF3j-NTA (Fig. 4), and showed that its disruption in yeast eliminated j/HCR1-association with the MFC *in vivo* (Fig. 7). This interaction is driven by conserved charge complementarity between the subunits and an evolutionary conserved hydrophobic pocket on the backside of the heIF3b-RRM, which accommodates the strictly conserved Trp residue in the heIF3j-NTD (Fig. S3). This recognition mode is also employed by the UHM family (U2AF homology motif) of non-canonical RRMs, which mediate protein-protein interactions through a conserved Arg-X-Phe motif in the L5 loop and a negatively charged, extended helix α1. UHM-ligand complexes share the crucial role of a conserved Trp residue from the ligand buried in a hydrophobic RRM pocket at the center of the protein interface as in case of the heIF3b-RRM - heIF3j complex 37; 38; 39 suggesting a general mode of protein recognition by these non-canonical RRMs (Fig. 4).

### eIF3j Contributes to the Delicate Process of Setting the Reading Frame for Decoding in co-operation with its conserved binding partner eIF3b-RRM and eIF1A

In this study, we presented two unexpected findings regarding the role(s) of the j/HCR1 subunit of eIF3 in translation: i) its NTD is sufficient to fulfill all functions of j/HCR1 needed to support wt growth of yeast cells (Fig. 1E); and ii) j/HCR1 is required for maintaining the proper control over the AUG start codon selection in co-operation with its binding partner b/PRT1 and eIF1A (Fig. 3) implying that it most likely stays ribosome-bound beyond mRNA recruitment at least to the point of AUG recognition.

Consistent with the placement of the heIF3j-CTD to the mRNA entry channel and ribosomal A site^27^, our *in vitro* binding assays revealed specific interactions between the CTD of j/HCR1 and RPS2 and RPS23 depending on its KERR motif (Fig. 1B and C). RPS23 is situated on the interface side under the A site, whereas RPS2 lies on the solvent side at the entry pore of the mRNA channel (Fig. 1D) 28. Placing the CTD of j/HCR1 into the mRNA entry channel suggests that the NTD of j/HCR1 most probably resides at the entry pore on the 40S solvent side, where the main body of eIF3 is thought to reside and thus where it could interact with the RRM of b/PRT1 (Fig. 1A) 14; 16. (The RRM of b/PRT1 interacts with the C-terminal part of the a/TIF32 subunit 10, which is also believed to occur near the entry pore of the mRNA binding track based on its previously reported interactions with helices h16-18 and RPS0A 8; 14.)

**Figure 8:**
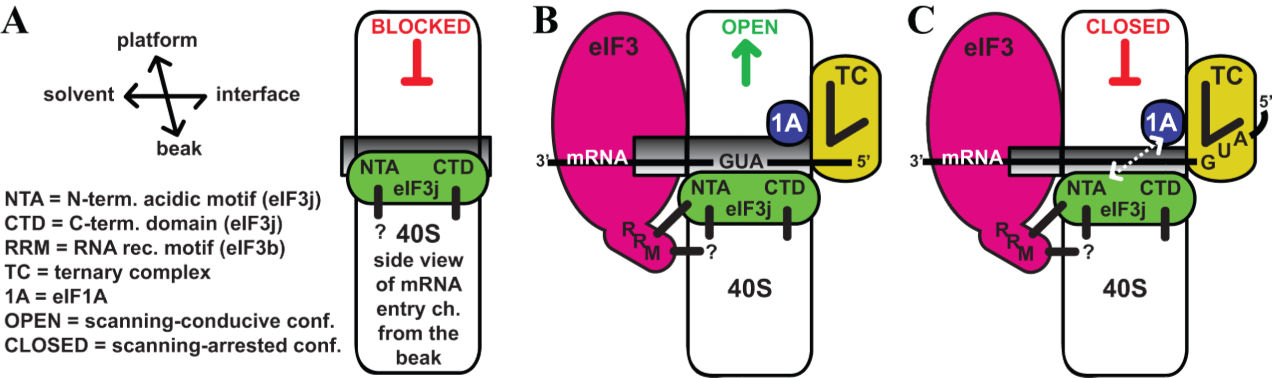
eIF3j/HCR1 co-operates with eIF3b/PRT1 and eIF1A to ensure stringent selection of the AUG start codon. (A) In the absence of eIFs, eIF3j/HCR1 occupies the mRNA entry channel to at least partially block mRNA recruitment. (B) Recruitment of TC and eIF3 that interacts with the NTA of eIF3j/HCR1 via the RRM of eIF3b/PRT1 clears the mRNA entry channel so that the ribosome can adopt the open, scanning-conducive conformation for mRNA recruitment. (C) Upon AUG recognition, eIF3j/HCR1 in cooperation with the eIF3b/PRT1-RRM functionally interacts with eIF1A to stimulate prompt switching to the closed, scanning-arrested conformation. Black thick lines represent direct interactions; dotted line with arrowheads indicates functional interaction between eIF3j/HCR1 and eIF1A.

Given its specific location and its observed negative co-operativity with mRNA in 40S-binding 27, heIF3j was predicted to regulate access of the mRNA-binding cleft and influence mRNA-40S subunit association during scanning and AUG recognition 27. Our results showing that deletion of *j/HCR1* or of its NTD produces a severe leaky scanning defect (Fig. 3C and D) are in prefect agreement with this prediction and suggest that eIF3j may contribute to stabilization of the properly formed pre-initiation complexes at the start codon. A similar role in pausing scanning upon establishment of the correct initiation codon-anticodon base-pairing was proposed for eIF1A 30. Interestingly, heIF3j showed negative co-operativity in 40S-binding also with eIF1A 27, and we indeed observed that the leaky scanning phenotype was partially (by ~ 50%) suppressed by overexpressing eIF1A (Fig. 3E). Furthermore, we found that destroying the specific contact between the j/HCR1-NTA and b/eIF3b-RRM by the *NTA1* and *α1*+*L5* mutations, respectively, also greatly increased leaky scanning phenotype, although not to the same extent as the deletion of the entire NTD (Fig. 3 and 7). Hence it is conceivable that other regions of the NTD of j/HCR1 are further required for the wt function. Given the fact that the RRM of b/PRT1 but not j/HCR1 play a critical role in stable eIF3 association with the 40S subunit (see below), these results strongly suggest that the major role of the evolutionary conserved interaction between eIF3j and eIF3b is to prevent skipping the proper AUG start codon during scanning. Based on these observations we propose the following model (Fig. 8).

Both terminal domains of yeast j/HCR1 make independent but synergistic interactions with the region on the 40S subunit including the 40S mRNA entry channel to at least partially block mRNA recruitment (Fig. 8A). It was shown that the negative co-operativity between heIF3j and mRNA is neutralized upon TC recruitment to the P site, even though heIF3j remains in the mRNA-binding cleft 27. Hence we further propose that the recruitment of TC with other eIFs including eIF3 may act together to clear the entry pore for mRNA recruitment, perhaps partially via establishment of the j/HCR1-NTA – b/PRT1-RRM interaction (Fig. 8B). Upon commencement of scanning, eIF3j/HCR1 in co-operation with eIF3b/PRT1-RRM makes most probably indirect functional contact with eIF1A that could influence the conformation and activity of eIF1A in helping to decode the initiation codon in a way that would prevent leaky scanning, possibly by prompt switching to the scanning-arrested conformation when the start codon has entered the P site (Fig. 8C).

j/HCR1 was previously shown to stimulate 40S-binding by eIF3 *in vivo* 12 and its human orthologue *in vitro* 7; 20; 26. Our *in vivo* formaldehyde crosslinking experiments (Fig. 2) combined with unpublished *in vitro* 40S – eIF3±j binding data from J. Lorsch’s lab (J. Lorsch, personal communication, 2009), however, suggest that this stimulatory activity of j/HCR1 might not be as strong as initially thought. With respect to this, the strong requirement of heIF3j for bringing purified eIF3 to the 40S subunit seems to indicate that yeast and human j subunits differ in the extent of this stimulation. Nevertheless, given the fact that the heIF3j requirement for 40S-binding by eIF3 was suppressed by the TC, eIF1, eIF1A or single stranded RNA or DNA co-factors 7; 26, the physiological significance of these *in vitro* observations with heIF3j will require careful examination in the living mammalian cells.

Unlike the *j/hcr1-NTA1* mutation, mutating the conserved hydrophobic pocket residues in *b/prt1-α1*+*L5* dramatically reduced 40S-occupancy by eIF3 and its associated eIFs *in vivo* (Fig. 7E). These findings strongly indicate that this activity of the b/PRT1-RRM region comprising the hydrophobic pocket is independent of its contact with the NTA of j/HCR1. Hence we propose that the RRM features α1 and L5, in addition to preventing the leaky scanning by interacting with the j/HCR1, most likely also form an important intermolecular bridge between eIF3 and the 40S subunit (Fig. 8B), such as that created by the NTD of a/TIF32 and RPS0a 8; 14.

Finally, it is noteworthy that the expression of the j/hcr1-NTD or CTD alone suppressed the 40S biogenesis defect of *j/hcr1Δ* cells 25 only partially (S.W. and L.V., unpublished observations) implying that the full-length j/HCR1 is needed for optimal function. Since the j/hcr1-NTD fully supports wt growth, we find it highly unlikely that the 40S biogenesis defect significantly contributes to *j/hcr1Δ* growth defects.

## MATERIALS AND METHODS

### Construction of Yeast Strains and Plasmids

To create SY73, H428 was introduced with YEp-j/hcr1-NTA1 and the resulting transformants were selected on media lacking leucine.

YAH06 was generated by a genetic cross of H426 (Table II) and H428 (*MAT*a *PRT1 leu2-3, 112 ura3-52 hcr1Δ*) 12. After tetrad dissection, spores with the slow growth phenotype suppressible by *j/HCR1*, resistant to 3-AT, unable to grow on 5-FOA and autotrophic for Tryptophan, were selected.

List of all PCR primers named below can be found in Table S1.

pGEX-j/hcr1-NTD was made by inserting the *Bam*HI-*Sal*I digested PCR product obtained with primers AD GST-HCR1 and AH-GSTHCR1-NTD-R using the template pGEX-j/HCR1 into *Bam*HI-*Sal*I digested pGEX-5X-3.

pGEX-j/hcr1-CTD was made by inserting the *Bam*HI-*Sal*I digested PCR product obtained with primers AH-GSTHCR1-CTD and AD GST-HCR1-R using the template pGEX-j/HCR1 into *Bam*HI-*Sal*I digested pGEX-5X-3.

pGEX-j/hcr1-NTA1 was made by inserting the *Bam*HI-*Sal*I digested PCR product obtained with primers AD GST-HCR1 and AD GST-HCR1-R using the template YEp-j/hcr1-NTA1 (see below) into *Bam*HI-*Sal*I digested pGEX-5X-3.

pT7-b/prt1-rrm-α1+L5 was made by inserting the *Nde*I-*Hind*III digested PCR product obtained with primers LVPNDEI-724 and LVPC136-724 using the template pRS-b/prt1-L5+α1-His (see below) into *Nde*I-*Hind*III digested pT7-7 40.

pGEX-j/hcr1-BOX9 was made by inserting the *Bam*HI-*Sal*I digested PCR product obtained with primers AD GST-HCR1 and AD GST-HCR1-R using the template YEp-j/hcr1-BOX9 (see below) into *Bam*HI-*Sal*I digested pGEX-5X-3.

pGEX-j/hcr1-Δ80 was made by inserting the *Bam*HI-*Nco*I digested PCR product obtained with primers AD GST-HCR1 and HCR1-80-NcoI-R using the template YEp-j/HCR1-DS into *Bam*HI-*Nco*I digested pGEX-5X-3.

pGEX-RPS2 was made by inserting the *Bam*HI-*Sal*I digested PCR product obtained with primers RPS2-f and RPS2-r using the template pGBKT7-RPS2 14 into *Bam*HI-*Sal*I digested pGEX-5x-3.

pGBK-T7-RPS23 was made by inserting the *Bam*HI-*Pst*I digested PCR product obtained with primers RPS23-f and RPS23-r using the template pGBKRPS23 14 into *Bam*HI-*Pst*I cleaved pGBKT7 (Novagen).

To construct pRS-b/PRT1-HisXS, the following pair of primers was used with pRSPRT1-His-LEU 12 as a template: AH-PRT1-BamHI and AH-PRT1-NotI-R. PCR product thus obtained was digested with *BamH*I-*Not*I and inserted into *BamH*I-*Not*I cleaved pRSPRT1-His-LEU. This subcloning step was done to remove the second *Xba*I and *Spe*I sites immediately following the stop codon of *b/PRT1* to facilitate subcloning of the RRM mutants.

pRS-b/prt1-L5+α1-His was constructed in two steps. First, the following two pairs of primers were used with pRS-b/PRT1-HisXS as a template: AH-PRT1-ApaI and LV-RRM-AALA-R; and LV-RRM-AALA-R and AH-PRT1-XbaI-R, respectively. The PCR products thus obtained were used in a 1:1 ratio as templates for a third PCR amplification with primers AH-PRT1-ApaI and AH-PRT1-XbaI-R. The resulting PCR product was digested with *Apa*I-*Xba*I and inserted into *Apa*I-*Xba*I cleaved pRS-b/PRT1-HisXS producing pRS-b/prt1-AALA-His. In the second step, pRS-b/prt1-AALA-His was used as a template for PCR with the following two pairs of primers: AH-PRT1-ApaI and AH-PRT1-A1B-R; and AH-PRT1-A1B and AH-PRT1-XbaI-R respectively. The PCR products thus obtained were used in a 1:1 ratio as templates for a third PCR amplification with primers AH-PRT1-ApaI and AH-PRT1-XbaI-R. The resulting PCR product was digested with *Apa*I-*Xba*I and inserted into *Apa*I-*Xba*I cleaved pRS-b/PRT1-HisXS.

YEp-j/HCR1-DS was constructed using the QuikChange® Multi Site-Directed Mutagenesis Kit from Stratagene according to the vendors instructions. In step 1, PCR was performed with the kit-provided enzyme blend using primers DS HCR1-BHI and DS HCR1-NcoI and YEpHCR1 24 as a template. This subcloning step was done to introduce the *Bam*HI site immediately preceding the AUG start codon and the *Nco*I sites immediately following the stop codon of *j/HCR1* to facilitate subcloning the j/HCR1 mutants.

YCp-j/HCR1-DS-U was constructed by inserting the 1289-bp *Hind*III-*Sac*I fragment from YEp-j/HCR1-DS into YCpLVHCR1-U 24 digested with *Hind*III-*Sac*I.

YEp-j/HCR1-DS-U was constructed by inserting the 1289-bp *Hind*III-*Sac*I fragment from YEp-j/HCR1-DS into YEplac195 41 digested with *Hind*III-*Sac*I.

YEp-j/hcr1-BOX9 was generated by fusion PCR. The following pairs of primers were used for separate PCR amplifications using YEp-j/HCR1-DS as template: (1) DS HCR1-BHI and AH-HCR1-BOX+9-R, respectively, (2) AH-HCR1-BOX+9 and AH-HCR1-NcoI-R, respectively. The PCR products thus obtained were used in a 1:1 ratio as templates for a third PCR amplification using primers DS HCR1-BHI and AH-HCR1-NcoI-R. The resulting PCR product was digested with *Bam*HI and *Nco*I and ligated with *Bam*HI-*Nco*I-cleaved YEp-j/HCR1-DS (replacing wt *j/HCR1* with *j/hcr1-BOX9*).

YEp-j/hcr1-NTA1 was generated by fusion PCR. The following pairs of primers were used for separate PCR amplifications using YEp-j/HCR1-DS as template: (1) DS HCR1-BHI and HCR1-NTA4-R, respectively, (2) SW-HCR1-NTA2+4 and AH-HCR1-NcoI-R, respectively. The PCR products thus obtained were used in a 1:1 ratio as templates for a third PCR amplification using primers DS HCR1-BHI and AH-HCR1-NcoI-R. The resulting PCR product was digested with *Bam*HI and *Nco*I and ligated with *Bam*HI-*Nco*I-cleaved YEp-j/HCR1-DS (replacing wt *j/HCR1* with *j/hcr1-NTA1*).

YEp-j/hcr1-NTA1-U was constructed by inserting the 1289-bp *Hind*III-*Sac*I fragment from YEp-j/hcr1-NTA1 into YEplac195 41 digested with *Hind*III-*Sac*I.

YEp-j/hcr1-NTD was constructed in two steps. First, the 817 bp insert obtained by digestion of pGEX-j/hcr1-NTD with *Bam*HI and *Not*I was ligated into *Bam*HI-*Not*I cleaved pRS303 42. The resulting plasmid was then cut with *Bam*HI-*Sac*I and the insert containing *j/hcr1-NTD* was used to replace full length *j/HCR1* in the *Bam*HI-*Sac*I cut YEp-j/HCR1-DS.

YEp-j/hcr1-CTD was made by inserting the *Bam*HI-*Nco*I digested PCR product obtained with primers AH-GST-HCR1-CTD and AH-HCR1-NcoI-R using YEp-j/HCR1-DS as a template into *Bam*HI-*Nco*I cut YEp-j/HCR1-DS (replacing wt *j/HCR1* with *j/hcr1-CTD*).

YEp-j/hcr1-NTD-NTA1 was made by inserting the *Bam*HI-*Nco*I digested PCR product obtained with primers DS HCR1-BHI and SW HCR1-NTD-NcoI-R using YEp-j/hcr1-NTA1 as a template into *Bam*HI-*Nco*I cut YEp-j/HCR1-DS (replacing wt *j/HCR1* with *j/hcr1-NTD-NTA1*).

### Yeast Biochemical Methods

GST pull-down experiments with GST fusions and *in vitro*-synthesized ^35^S-labeled RPS2, RPS23a, j/hcr1-NTD, j/hcr1-CTD and b/prt1-RRM polypeptides (see Table III for vector descriptions) were conducted as follows. Individual GST-fusion proteins were expressed in *E. coli*, immobilized on glutathione-Sepharose beads and incubated with 10 μl of ^35^S-labeled potential binding partners at 4°C for 2 h. The beads were washed 3 times with 1 ml of phosphate-buffered saline and bound proteins separated by SDS-PAGE. Gels were first stained with Gelcode Blue Stain Reagent (Pierce) and then subjected to autoradiography. (GST-RPS23 could not be tested due its insolubility in bacterial lysates.) Ni^2+^-chelation chromatography of eIF3 complexes containing His-tagged b/PRT1 from yeast whole-cell extracts (WCEs) and Western blot analysis were conducted as described in detail previously 43. In short, WCEs were incubated with 4 μL of 50% Ni2+-NTA-silica resin (Qiagen) suspended in 200 μL of buffer A for 2 h at 4°C, followed by washing and elution. Fractionation of native pre-initiation complexes in WCEs from HCHO cross-linked cellsthrough sucrose gradients, including resedimentation analysis, were carried out according to 29.

### NMR Spectroscopy

NMR experiments were performed on Bruker AMX500 or AVANCE800 spectrometers equipped with cryoprobes and on a Bruker DMX600 spectrometer. ^1^H, ^13^C, and ^15^N Chemical shifts assignment was achieved by means of through-bond heteronuclear scalar correlations with standard pulse sequences recorded on either ^13^C/^15^N-labeled heIF3b-RRM complexed with the heIF3j peptide or on ^13^C/^15^N-labeled heIF3j peptide complexed with heIF3b-RRM in NMR buffer (20 mM deuterated-TRIS (pH 7.5) and 100 mM NaCl) containing 10% ^2^H_2_O. Acquisition of NOEs was accomplished using a series of standard 3D heteronuclear experiments. Intermolecular NOEs between the heIF3b-RRM domain and the heIF3j peptides (long = residues 1-69 of heIF3j with a deletion of 6 out of 7 non-conserved N-terminal alanine residues according to 18 or short = residues 35-69 of heIF3j) were obtained from 2D and 3D _13_C-filtered NOESY experiments recorded on _13_C/_15_N-labeled heIF3b-RRM complexed with the long or short heIF3j peptide and on the long or short _13_C/_15_N-labeled heIF3j peptide complexed with heIF3b-RRM in a 100% _2_H_2_O solution. Comparison of the intermolecular NOE pattern for the short and long heIF3j peptides revealed no significant differences and more importantly, no additional NOEs could be observed with the longer peptide. Therefore, the heIF3b-RRM complex with the shorter heIF3j peptide was chosen for a high-resolution structure determination. All NMR samples were prepared in 20 mM deuterated-TRIS (pH 7.5) and 100 mM NaCl. Concentrations were 0.7 mM for heIF3b-RRM domain with the heIF3j peptides added at a concentration of 0.7-1.0 mM in order to saturate the heIF3b-RRM domain with the long or short heIF3j peptide. All spectra were recorded at 25°C.

### Structure Calculation

The structure of the heIF3b-RRM/heIF3j_35-69_ peptide complex was calculated using the program CYANA 44. 1853 NOE-based distances derived from 3D heteronuclear NOESY experiments as well as 113 dihedral angle restraints (Φ and ψ) obtained by analysis of N, Hα, Cα, and C_β_ chemical shift values using the TALOS program 45; 46 were used in the structure calculations. A total of seven iterations for structural calculations and distance restraint assignment were run with CYANA. 100 structures were calculated, and the 10 structures having the lowest energies were adopted. These structures were then water refined in a minimization run using the SANDER module of AMBER 9.0 47. The quality of each structure was assessed using the program Procheck-NMR 48. A list of all restraints and structural statistics is presented in Table 1. Figures were prepared using the programs PyMOL (http://pymol.sourceforge.net/) and MOLMOL 49.

### The NMR Structure Determination of the heIF3b-RRM_170-274_ - heIF3j_35-69_ Complex

The N-terminal heIF3j_35-69_ fragment of heIF3j displays the same binding mode as both full-length heIF3j and the larger N-terminal heIF3j_1-69_ peptide displaying very similar chemical shift perturbations in heIF3b-RRM 18 (Fig. S2A). More importantly, the same binding mode of both N-terminal heIF3j fragments was evidenced by virtually identical intermolecular NOEs of eleven residues surrounding Trp52 (Fig. S2B). The structure of the complex was solved using 1916 experimental restraints that consist of 1853 distance restraints derived from Nuclear Overhauser Effect (NOE) data including 32 intermolecular NOEs extracted from isotope-filtered 2D and 3D experiments. In addition, 113 dihedral angle restraints (ϕ and ψ angle restraints) were included from the analysis of ^13^C_α/β_ chemical shifts using the program TALOS 46). Out of 100 calculated structures, the 10 lowest-energy structures having the best agreement with experimental restraints were subsequently refined in explicit solvent to improve the local geometry, electrostatics, and packing quality for the complex. Stereo views of the 10 lowest-energy structures (Fig. S1) and the structural statistics (Table 1) demonstrate a well-defined complex structure with low pairwise rmsd values of 1.19 ± 0.4 Å for heavy atoms corresponding to residues 180-266 and 45-55 of heIF3b-RRM and heIF3j, respectively.

### Preparation of Human Proteins

His-tagged heIF3b-RRM domain and heIF3j subunit were constructed as described previously 18 and transformed in *E. coli* BL21(DE3) cells. Cultures for heIF3b-RRM, heIF3j and their mutants were grown at 37°C, and protein over-expression was induced by addition of 1 mM of IPTG at A_600_ 0.8. Cells were harvested 3 hours after induction. For isotope labeling, minimal media containing ^15^NH_4_Cl and ^13^C-glucose were used. All protein samples were purified over a nickel-chelating column (HiTrap, Amersham Biosciences), and this was followed by TEV protease cleavage for His-tag removal. The reaction mixture was then reloaded on a HiTrap chelating column charged with nickel sulfate to remove all of the TEV protease, the His-tag as well as minor contaminating proteins. After purification, the proteins were exchanged to appropriate buffer for subsequent experiments and further concentrated.

### Preparation of heIF3j Peptide

A DNA fragment encoding the heIF3j peptide sequence (residues 35-69) was prepared by PCR from full-length heIF3j plasmid DNA, digested with *Nde*I and *Eco*RI and ligated into a modified pET28a vector (Novagen, containing an N-terminal His_6_-tag fused to a lipoyl domain 50 followed by a TEV cleavage site and the standard pET28a multiple cloning site) digested with the same enzymes. *E. coli* BL21(DE3) cells were transformed with the heIF3j peptide construct and grown at 37°C in rich LB medium or minimal media containing ^15^NH_4_Cl and ^13^C-glucose for production of unlabeled or labeled peptide, respectively. Protein over-expression was induced by addition of 1 mM IPTG at A_600_ 0.8. The heIF3j peptide fused to lipoyl domain was purified over a nickel-chelating column. TEV protease was then used to separate the heIF3j peptide from the lipoyl domain. Isolation of the heIF3j peptide required loading on a nickel-chelating column. This was followed by ion exchange (HiTrap DEAE, Amersham Biosciences) for further purification of the peptide.

### ITC Experiments

All calorimetric titrations were performed on a VP-ITC microcalorimeter (Microcal). Protein samples were extensively dialyzed against the ITC buffer containing 20 mM Hepes (pH 7.5), 200 mM NaCl. All solutions were filtered using membrane filters (pore size 0.2 μm) and thoroughly degassed for 20 min by gentle stirring under argon. The sample cell was filled with 50 μM solution of full-length heIF3j wt or mutants and the injection syringe with 1 mM of the titrating heIF3b-RRM. Each titration typically consisted of a preliminary 2.5 μl injection followed by 58 subsequent 5μl injections every 210 seconds. All of the experiments were performed at 25 °C. Data for the preliminary injection, which are affected by diffusion of the solution from and into the injection syringe during the initial equilibration period, were discarded. Binding isotherms were generated by plotting heats of reaction normalized by the moles of injectant versus the ratio of total injectant to total protein per injection. The data were fitted using Origin 7.0 (Microcal).

### Pull-down Experiments

His_6_-tagged heIF3b-RRM (wt and mutants) and untagged full-length heIF3j subunit were prepared as described above and buffer-exchanged in equilibration buffer (50 mM sodium phosphate pH 8, 100 mM NaCl). Each His^6^-heIF3b-RRM construct was incubated with unlabeled at heIF3j (30μM final concentration of each protein) for 15 min at room temperature and loaded on His-select spin columns (Sigma) equilibrated with equilibration buffer. After two washing steps with equilibration buffer containing 5 mM imidazole, proteins were eluted with elution buffer (50 mM sodium phosphate pH 8, 100 mM NaCl, 250 mM imidazole). The eluted proteins were resolved by denaturating gel electrophoresis and visualized by staining with InstantBlue (Novexin). Percentage of heIF3j bound fraction was evaluated by measuring band intensities with ImageJ program.

## Supplementary Material

01

## Figures and Tables

**Table 1:** Structural statistics of the heIF3b-RRM_170-275_ - heIF3j_35-69_ complex^a^

Restraints Used for Structure Calculations	
Total NOE distance restraints	1853
Short range (intraresidue)	966
Medium-range (1<|i-j|<5)	289
Long-range (|i-j|>=5)	566
Intermolecular	32
Dihedral angle restraints (ϕ, ψ)	113

Structural Statistics	
Ramachandran plot^b^ (%)	
Residues in the most favored regions	71.4
Residues in additional allowed regions	27.0
Residues in generously allowed regions	1.3
Residues in disallowed regions	0.4

r.m.s. deviations of atomic coordinates (Å)	
heIF3b-RRM_184-268_ - heIF3j_45-55_ complex	
Backbone atoms	0.726
All heavy atoms	1.192

a The 10 conformers with the lowest energies were selected for statistical analysis. Because of the absence of medium range, long range and intermolecular NOEs involving residues 35-44 and 58-69 of heIF3j_35-69_, these residues were not included in the calculations.

b Based on PROCHECK-NMR analysis.

**Table 2:** Yeast strains used in this study

Strain	Genotype	Source or reference
H416^a^	*MATa leu2-3,112 ura3-52*	12
H417^a^	*MATa leu2-3,112 ura3-52 trp1Δ*	11
H425^a^	*MATa leu2-3,112 ura3-52 trp1Δ b/prt1::hisG* *gcn2::hisG (lc b/PRT1 URA3)*	12
H428^a^	*MATa leu2-3, 112 ura3-52 j/hcr1Δ*	12
SY73^a^	*MATa leu2-3, 112 ura3-52 j/hcr1Δ (hc j/hcr1*- *NTA1 LEU2)*	This study
YAH06	*MATa leu2-3,112 ura3-52 trp1Δ b/prt1::hisG* *GCN2 (lc b/PRT1 URA3)*	This study
H3674^a^	*MATa leu2-3, 112 ura3-52 b/prt1-rnp1*	12

a Isogenic strains.

**Table 3:** Plasmids used in this study

Plasmid	Description	Source of reference
pGEX-5X-3	cloning vector for GST fusions	51
pGEX-j/HCR1	GST-j/HCR1 fusion plasmid from pGEX-5X-3	23
pGEX-j/hcr1-NTD	GST-j/hcr1-NTD [1-135] fusion plasmid from pGEX-5X-3	This study
pGEX-j/hcr1-CTD	GST-j/hcr1-CTD [136-265] fusion plasmid from pGEX-5X-3	This study
pGEX-j/hcr1-NTA1	GST-j/hcr1-NTA1 fusion plasmid from pGEX-5X-3	This study
pT7-b/PRT1-RRM (ΔA)	*b/PRT1*[1-136] ORF cloned under T7 promoter	23
pT7-b/prt1-rrm-α1+L5	*b/PRT1*[1-136] ORF containing the *α1+L5* mutation cloned under T7 promoter	This study
pGEX-j/hcr1-BOX9	GST-j/hcr1-BOX9 fusion plasmid from pGEX-5X-3	This study
pGEX-j/hcr1-Δ80	GST-j/hcr1-Δ80 [1-185] fusion plasmid from pGEX-5X-3	This study
pGBK-T7-RPS2	*RPS2* ORF cloned into pGBKT7, *TRP1* (Clontech)	14
pGBK-T7-RPS23	*RPS23* ORF (without intron) cloned into pGBKT7, *TRP1* (Clontech)	This study
pGEX-RPS2	GST-RPS2 fusion plasmid from pGEX-5X-3	This study
pRS-b/PRT1-HisXS	low copy wt *b/PRT1* in *LEU2* plasmid from pRS315	This study
pRS-b/prt1-L5+α1-His	low copy *b/PRT1* containing the *α1+L5* mutation in *LEU2* plasmid from pRS315	This study
YEplac181	high copy cloning vector, *LEU2*	41
YEplac195	high copy cloning vector, *URA3*	41
YEp-j/HCR1-DS	high copy *j/HCR1* wt coding region flanked by *Bam*HI and *Nco*I sites, respectively, in *LEU2* plasmid from YEplac181	This study
YCp-j/HCR1-DS-U	low copy wt *j/HCR1* in *URA3* plasmid from YCplac33	This study
YEp-j/HCR1-DS-U	high copy *j/HCR1* wt coding region flanked by *Bam*HI and *Nco*I sites, respectively, in *URA3* plasmid from YEplac195	This study
YEp-j/hcr1-NTA1	high copy *j/HCR1* containing the NTA1 mutation in *LEU2* plasmid from YEplac181	This study
YEp-j/hcr1-NTA1-U	high copy *j/HCR1* containing the NTA1 mutation in *URA3* plasmid from YEplac195	This study
YEp-j/hcr1-NTD	high copy *j/hcr1-NTD* [1-135] in *LEU2* plasmid from YEplac181	This study
YEp-j/hcr1-CTD	high copy *j/hcr1-CTD* [136-265] in *LEU2* plasmid from YEplac181	This study
YEp-j/hcr1-NTD-NTA1	high copy *j/hcr1-NTD* [1-135] containing the NTA1 mutation in *LEU2* plasmid from YEplac181	This study
YEplac24	High copy cloning vector, URA3	52
p1780-IMT	high-copy *SUI2, SUI3, GCD11, IMT4, URA3* plasmid from YEp24	53
p180 (YCp50–GCN4–lacZ)	Low copy *URA3* vector containing wild-type *GCN4* leader	54
pM226	Derivative of pM199; ORF of uORF1 extends into the *GCN4-lacZ* coding region	32
plig102-3	Low copy *URA3* vector with *GCN4* leader point mutations containing uORF4 only at its original position in front of the *GCN4-lacZ* coding region	32
pDSO22	high-copy *TIF11* (eIF1A), *URA3* plasmid from YEplac195	55

## Abbreviations used

TC: ternary complex (eIF2/Met-tRNA_i_^Met^/GTP)
MFC: multifactor complex
eIF: eukaryotic translation initiation factor
RRM: RNA-recognition motif
NTD: N-terminal domain
CTD: C-terminal domain
NTA: N-terminal acidic motif
Gcn^−^: general control non-derepressible
wt: wild type
ORF: open reading frame
ITC: Isothermal Titration Calorimetry
3-AT: 3-aminotriazole
WCE: whole-cell extract
α1: helix α1
L5: loop 5
Slg^−^: slow growth
Ts^−^: temperature sensitive
hc TC: a plasmid overexpressing all three subunits of eIF2 and tRNA_i_^Met^
heIF3b: human eIF3b
heIF3j: human eIF3j
