# Supplementary material for: The indispensable N-terminal half of eIF3j/HCR1 co-operates with its structurally conserved binding partner eIF3b/PRT1-RRM and eIF1A in stringent AUG selection
Source: J Mol Biol. Author manuscript; Available in PMC 2010 Sep 21. (PMC2824034; doi:10.1016/j.jmb.2009.12.047)
Supplement: 01 [file NIHMS169901-supplement-01.pdf]

SUPPLEMENTARY DATA

SUPPLEMENTARY FIGURE LEGENDS

Figure S1. Stereo View of the NMR Ensemble of the heIF3b-RRM$_{170-274}$ - heIF3j$_{35-69}$ Complex Peptide Complex. The 10 lowest-energy structures of heIF3b-RRM (in grey and green) and heIF3j (in yellow) are shown. The structures were fit using the backbone atoms C', C^{α}, and N of residues 184-264 of heIF3b-RRM and residues 45-55 of heIF3j.

Figure S2. The NMR Data Spectra of the heIF3b-RRM$_{170-274}$ - heIF3j$_{35-69}$ Complex. (A) Overlay of ^{1}H-^{15}N HSQC spectra of free heIF3b-RRM (black), heIF3b-RRM bound to full-length heIF3j (blue), heIF3j$_{1-69}$ (red), and heIF3j$_{35-69}$ (green). Sample concentrations were 0.7 mM for heIF3b-RRM domain with the heIF3j peptides (heIF3j$_{1-69}$ or heIF3j$_{35-69}$) added up to a final concentration of 0.7-1.0 mM (see Material and Methods). Some of the peaks showing the largest chemical shift variations upon binding are shown in close-up views and labeled. Addition of each heIF3j constructs induced significant and very similar chemical shift changes. Full-length heIF3j and heIF3j$_{1-69}$ induced the stronger chemical shift changes compared to heIF3j$_{35-69}$ suggesting a weaker binding affinity of the shorter peptide. Nevertheless, the strongest perturbations are located in the same regions as seen for heIF3j and heIF3j$_{1-69}$. This shows that the molecular recognition of all three heIF3j constructs by heIF3b-RRM is similar. (B) Comparison of strip plots of ^{1}H-^{1}H planes extracted from 2D ^{13}C-filtered NOESY spectra recorded on the complex of the heIF3j peptides (heIF3j$_{1-69}$ in black, and heIF3j$_{35-69}$ in red) and ^{13}C/^{15}N-labeled heIF3b-RRM domain in a 100% $^{2}H_2O$ solution. 19 out of the 32 intermolecular NOE correlations between heIF3j and heIF3b-RRM used for structure calculations of the complex are annotated (primed number correspond to heIF3j resonances).

Figure S3. Amino Acid Sequence Alignment of the heIF3b-RRM and the heIF3j-NTA with Other Species. (A) The amino acid sequence of heIF3b-RRM is aligned with its Mus musculus (Mouse) homolog (accession number P55884), Rattus Norvegicus homolog (Rat) (accession number Q4G061), Dictyostelium discoideum (Dicdi) homolog (accession number Q54QW1), Arabidopsis thaliana (Arath) homolog (accession number Q9C5Z1), Nicotiana tabacum (Tobac) homolog (accession number P56821), Saccharomyces cerevisiae (Yeast) homolog (accession number P06103), and Schizosaccharomyces pombe (Schpo) homolog (accession number Q10425). The alignment was conducted with ClustalW2 (http://www.ebi.ac.uk/Tools/clustalw2/index.html). Identical residues are shown with a dark background, whereas similar residues are shown with a lighter background. Corresponding secondary structures and the loop L5 of heIF3b-RRM are shown on top of the sequence. At the bottom of the sequence, residues presenting intermolecular noes used in structure calculations are highlighted with a star, and residues highlighted with circles are at a distance < 5Å of any atom of the protein partner. (B) The amino acid sequence of the heIF3j-NTA is aligned with its Mus musculus (Mouse) homolog (accession number Q66JS6), Rattus Norvegicus homolog (Rat) (accession number A0JPM9), Dictyostelium discoideum (Dicdi) homolog (accession number Q54KI0), Saccharomyces cerevisiae (Yeast) homolog (accession number Q05775), Bos taurus (Bovin) homolog (accession number Q0VCU8), and Pongo abelii (Ponab) homolog (accession number Q5R8D1).

Table S1. Oligonucleotides used in this study.

Oligonucleotide	Sequence (5' to 3')
AD GST-HCR1	AATTAGGATCCATATGTCTTGGGACGACG
AH-GSTHCR1-NTD-R	ATATATGTCGACTTAAGGTGTGTCCTTGG
AH-GSTHCR1-CTD	TATAAGGATCCATATGATCGAAACACATC
AD GST-HCR1-R	AATAAGTCGACTTACATAAAGTCGTCATC
DS HCR1-BHI	CAAAAAAAAAAAAGTAATAAAAGGATCCATGTCTTGGGACGACGA
AH-HCR1-NcoI-R	AGCAGATTACCATGGCATAAAGTC
AH-HCR1-BOX+9	CAAACAGTTGCTACTTTGAACGTTTTGATCGCAGCTGCTGCAGCAGCAGCAGCAGCAGCAGCAC TGGCCCGTGTTAGAGGTGGT
AH-HCR1-BOX+9-R	GATCAAAACGTTCAAAGTAGCAACTGTTTG
HCR1-80-NcoI-R	ATAGAGCTCCATGGTGCTACATCTCTAA
SW HCR1-NTD-NcoI-R	TATATACCATGGAGGTGTGTCCTTGGTAAAGGC
SW-HCR1-NTA2+4	GAAATTGGTGATGACGAACCTGCGATGGCTTCAGCACGTGCACGTCGACGTCGTAAGAAGCCTG CCCCAAAGCCA
HCR1-NTA4-R	CGCAGGTTCGTCATCACCAATTTC
AH-PRT1-BamHI	TCACTTGGGATCCATCTGGTA
AH-PRT1-NotI-R	ATATGCGGCCGCGGATCTTTAGTGGTGGTGGTGGTGGTGGT

AH-PRT1-ApaI	GGTACCGGGCCCCCCCTC
AH-PRT1-XbaI-R	AGTTCTCTCTAGATTCCACCA
LV-RRM-AALA	GGTAAAAGACTGGATTTAAAAGCAGCTTTGGCACTTTATACTATGAAAGATGTT
LV-RRM-AALA-R	TGCTTTTAAATCCAGTCTTTTACC
AH-PRT1-A1B	GTTTTGAAAAAGGCTTTGACTTCTGCAGCAGCTGAAGCTGGTAAAGTTGTTAACATGGAA
AH-PRT1-A1B-R	AGAAGTCAAAGCCTTTTTCAAAAC
LVPNDEI-724	CCACGCATATGACTACCGAGACTTTCGAA
LVPC136-724	CCTAAAAGCTTGAATTCGGTGTCAAAGTCGTC
DS HCR1-NcoI	TTCGGTGATGACGACTTTATGCCATGGTAATCTGCTTATTGTTTCTTTGC
RPS23-f	GCGCGGATCCATATGGGTAAAGGTAAGCCAAGAGGTTTGAACTCTGCTAGAAAGCTACGTGTCC ACAGAAGAAACAACCGTTGGGCCGAAAACAACTACAAGAA
RPS23-r	CCCCTGCAGTTATGATCTTGGCTTTTCCTT

Elantak_FigS1

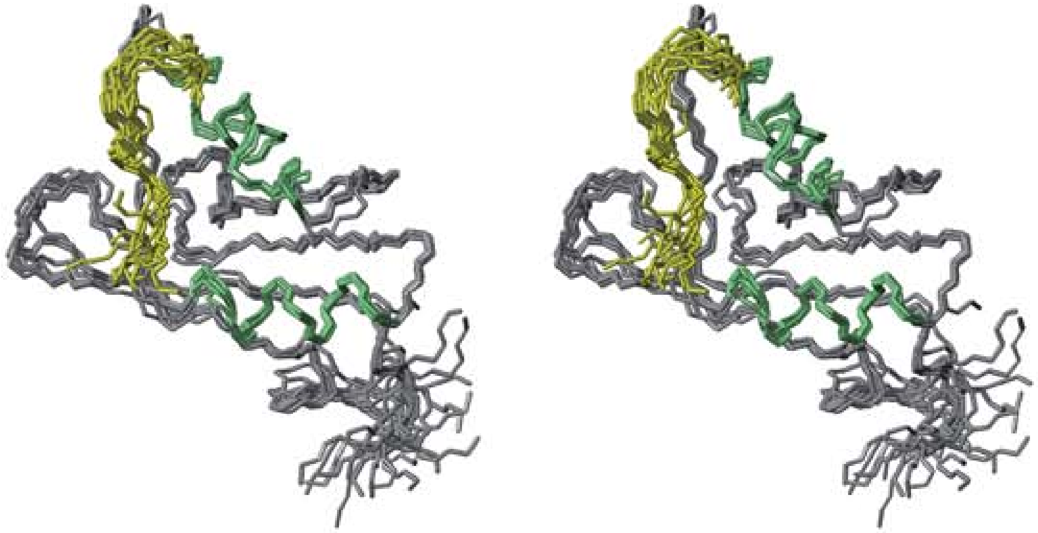

Elantak_FigS2

A

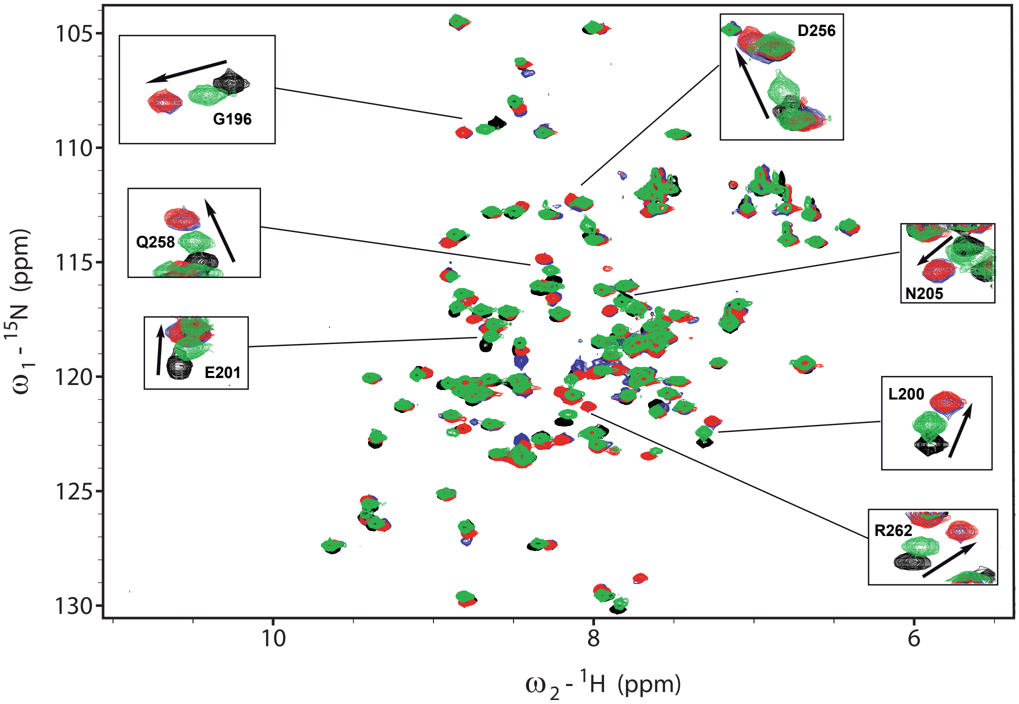

B

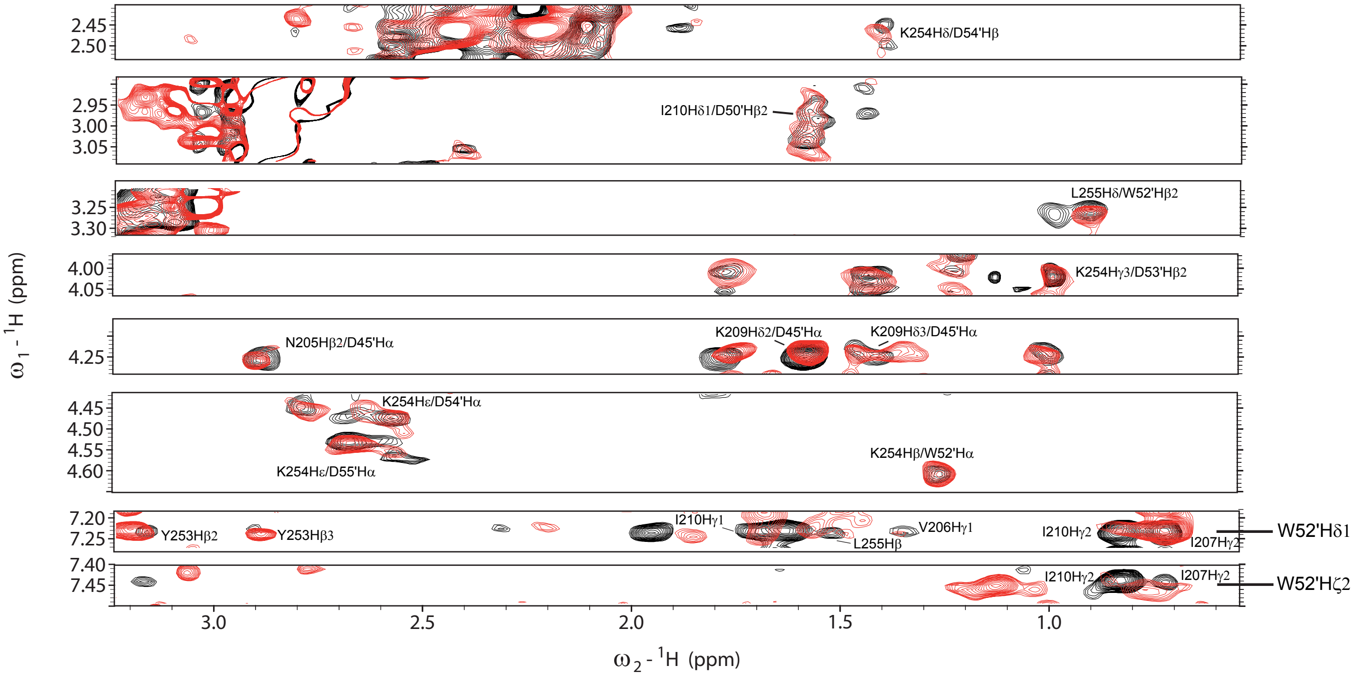

A

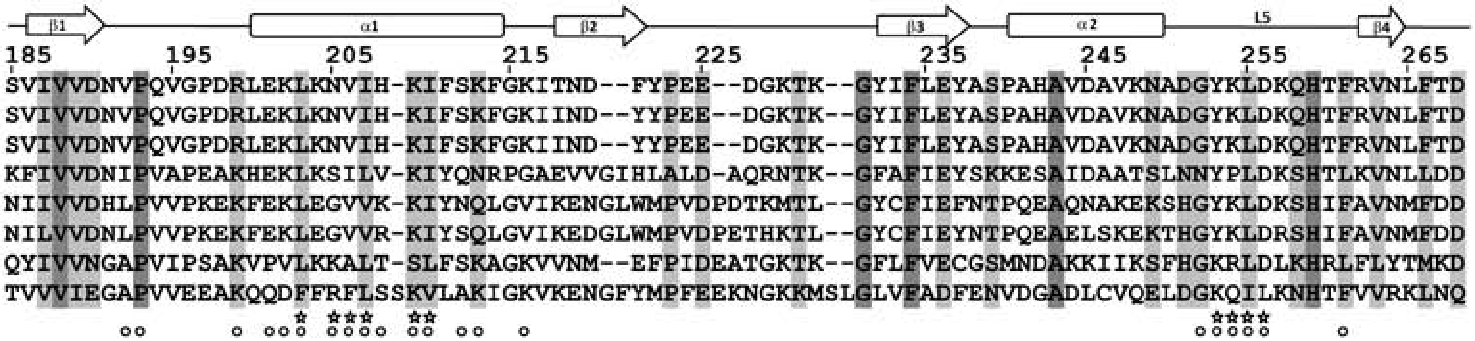


```
      185       195       205        215         225           235       245       255       265
Human SVIVVDNVPQVGPDRLEKLKNVIH-KIFSKFGKITND--FYPEE--DGKTK--GYIFLEYASPAHAVDAVKNADGYKLDKQHTFRVNLFTD
Mouse SVIVVDNVPQVGPDRLEKLKNVIH-KIFSKFGKIIND--YYPEE--DGKTK--GYIFLEYASPAHAVDAVKNADGYKLDKQHTFRVNLFTD
Rat   SVIVVDNVPQVGPDRLEKLKNVIH-KIFSKFGKIIND--YYPEE--DGKTK--GYIFLEYASPAHAVDAVKNADGYKLDKQHTFRVNLFTD
Dicdi KFIVVDNIPVAPEAKHEKLKSILV-KIYQNRPGAEVVGIHLALD-AQRNTK--GFAFIEYSKKESAIDAATSLNNYPLDKSHTLKVNLLDD
Arath NIIVVDHLPVVPKEKFEKLEGVVK-KIYNQLGVIKENGLWMPVDPDTKMTL--GYCFIEFNTPQEAQNAKEKSHGYKLDKSHIFAVNMFDD
Tobac NILVVDNLPVVPKEKFEKLEGVVR-KIYSQLGVIKEDGLWMPVDPETHKTL--GYCFIEYNTPQEAELSKEKTHGYKLDRSHIFAVNMFDD
Yeast QYIVVNGAPVIPSAKVPVLKKALT-SLFSKAGKVVNM--EFPIDEATGKTK--GFLFVECGSMNDAKKIIKSFHGKRLDLKHRLFLYTMKD
Schpo TVVVIEGAPVVEEAKQQDFFRFLSSKVLAKIGKVKENGFYMPFEEKNGKKMSLGLVFADFENVDGADLCVQELDGKQILKNHTFVVRKLNQ
                        ☆ ☆☆☆  ☆☆                                                ☆☆☆☆
             oo     o ooo oooo oo oo  o                                         ooooo    o
```

B

```
      35        45        55         65
Human GGDRWEGEDEDED-VKDNWDDDDDEKKEEAEVKPEV
Mouse GGDRWEGEDEDED-VKDNWDDDDDENKEEAEVKPEV
Rat   GGDRWEGEDEDED-VKDNWDDDDDENKEEAEVKPEV
Dicdi QKDKWINENREVE-EVDAWDASS----EDEVEKVEE
Bovin GGDRWEGEDEDED-VKDNWDDDDDEKKEEAEVKPEV
Ponab GGDRWEGEDEDED-VKDNWDDDDDEKKEEAEVKPEV
Yeast LMDSWDAEIGDDEPVMQSWDAEEEEKKPAPKPKKEQ
                ☆   ☆ ☆☆☆☆☆☆
              oooo  oooooooo
```
